# Evaluating the internalisation of the intrinsic role of health advocacy of student pharmacists in a new integrated Bachelor of Pharmacy curriculum: a mixed-methods study

**DOI:** 10.1186/s12909-023-04877-y

**Published:** 2023-11-27

**Authors:** Li Hui, Candice Lau, Jolin Xin Ni Wong, Julian Azfar, Paul John Gallagher, Leroy Koh

**Affiliations:** 1 Department of Pharmacy, National University of Singapore, Singapore, Singapore; 2 Saw Swee Hock School of Public Health, National University of Singapore, Singapore, Singapore

**Keywords:** Pharmacy, Education, Curriculum design, Integration, Spiral curriculum, Health advocacy, Healthcare disparities, Programme evaluation, Entrustable professional activities, Competency

## Abstract

To keep up with the contemporary health landscape, there is an imperative need for healthcare professionals to practise health advocacy through health promotion on the individual, population, and systems levels. In the Academic Year of 2020/2021, the National University of Singapore (NUS) Department of Pharmacy implemented a new spiral curriculum integrating basic, clinical, and systems sciences with one of its aims to deepen students’ health advocacy internalisation and prepare them as future health advocates. A mixed-methods approach was adopted. Questionnaires were disseminated across three time-points to elicit students’ levels of internalisation of health advocacy, which were then categorised into levels, and a Mann–Whitney U test was conducted. In comparison with prematriculation, no significant difference was found after students underwent the first year of the curriculum, while a significant difference was found after students underwent two years of the curriculum. Semi-structured interviews were also conducted after each Academic Year to gain deeper insights into the questionnaire results. Thematic analysis of the interviews revealed that curricular integration in the first year was perceived to be lacking. However, with learnt knowledge constantly reinforced and more experiential learning opportunities incorporated throughout the second year, students found the integrated curriculum beneficial in instilling confidence to practise health advocacy. This study offers insights into the prospects of a spiral integrated curriculum in imparting health advocacy, and may even suggest its potential to be applied to other educational settings. Future follow-up studies can also be conducted on the same study population to evaluate long-term impacts and areas for improvement of the curriculum.

**Supplementary Information:**

The online version contains supplementary material available at 10.1186/s12909-023-04877-y.

## Introduction

Health goes beyond healthcare, with most health problems stemming from upstream factors, including housing [1], education and diet [2]. Thus, it has become increasingly imperative to consider upstream social determinants of health together with downstream medical problems [3].

These rapidly evolving public health needs have been paralleled by an expansion of healthcare professionals’ roles to include the practice of health advocacy [4]. This involves promoting the health of individuals or populations, such as advocating for equal healthcare and reviewing health policies and institutional practices to reduce health disparities [5]. Considering its significance in providing holistic healthcare, health advocacy is now a core competency expected of healthcare professionals according to the widely adapted CanMEDS framework [6]. Pharmacists’ frequent interactions with patients render them well-positioned to advocate for health [7]. However, it remains challenging to empower pharmacists to address health disparity [8] as many believe that health advocacy is beyond their scope of practice [9].

To prepare future pharmacists to become health advocates and recognise its value, pharmacy education should holistically encompass medical concepts, clinical experience [10], and health advocacy concepts [11]. However, traditional pharmacy curricula are inadequate [12] as the public health sciences are taught independently in a separate module from the clinical sciences and experiential learning is introduced belatedly [13]. Subsequently, students fail to connect multidisciplinary concepts [14, 15]. and perceive health advocacy as a separate field from their clinical duties.

It is postulated that integrating basic, clinical, and systems sciences [11] in pharmacy curricula cultivates health advocacy by reframing the traditionally separately taught pillars into an interdependent framework [16, 17]. Integrated learning is paramount for students to extend beyond self-awareness of health advocacy, and to practise it on the individual, population, and ultimately, systems levels [18].

At the National University of Singapore (NUS) Department of Pharmacy, the longstanding curriculum was structured as a traditional block curriculum, which taught pharmaceutics, patient care, medicinal chemistry, physiological systems and other modules separately. In the Academic Year of 2020/2021 (AY20/21), the NUS Department of Pharmacy introduced an integrated, spiraled curriculum to transform the undergraduate program based on the CanMEDS framework [6], of which health advocacy is one of the competencies. Essentially, the new curriculum sought to iteratively reinforce intrinsic health advocacy concepts, allowing students to internalise its connection with various sciences. While various educational institutions worldwide have adopted integrated pharmacy curricula [19], there is a lack of evaluation of their impacts and much less on health advocacy outcomes. This study aims to address this gap by evaluating the internalisation of the intrinsic role of health advocacy after pharmacy students underwent the new integrated pharmacy curriculum at NUS.

## Methods

### Curriculum integration

In AY20/21, the NUS Pharmacy Class of 2024 was placed under the new integrated curriculum. Physiological systems formed the bedrock, and basic, clinical, and systems sciences were taught around it interconnectedly.

**Fig. 1:**
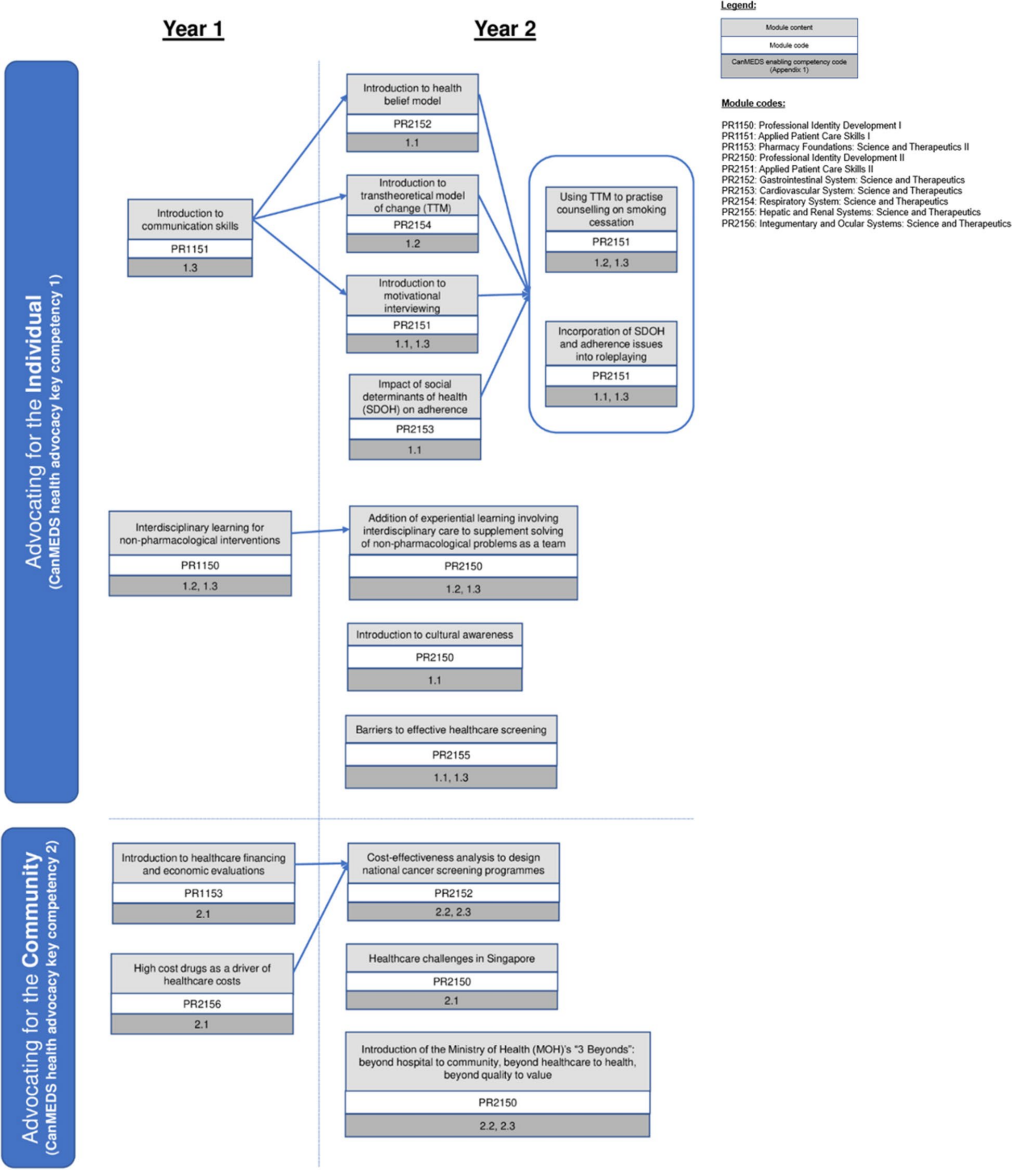
Health advocacy integration nodes throughout the new Bachelor of Pharmacy curriculum

Figure 1 shows how health advocacy concepts were integrated into modules and how they spiral in increasing complexity during the first two years of the curriculum.

In alignment with the CanMEDS framework [6], the curriculum aimed to train students to prevent and alleviate diseases by addressing social determinants of health, encouraging healthy lifestyle habits and through close health monitoring. These should be conducted for (1) individual patients by establishing partnerships with both patients and their families, and (2) communities by addressing system-level upstream factors of health inequity through improving clinical and institutional practices. The list of competencies can be found in Additional file 1: Appendix 1.

For example, through the PR2156 Integumentary and Ocular Systems (physiological system) module in Year 1, students would become cognisant of exponentially rising healthcare costs due to increasing drug prices (basic sciences) by leveraging on the theme of efficacious yet costly biologics (systems science involving drug cost-effectiveness), which is used for psoriasis treatment (clinical science). In Year 2, the Transtheoretical Model of Change was introduced to explore individual behavioural change (basic science) during the topic of smoking cessation (systems sciences as part of health promotion activities) in asthma exacerbation prevention (clinical science) in PR2154 Respiratory Systems (physiological system).

A spiral integrated approach [20] should enhance health advocacy understanding by (1) incorporating the concept in progressive difficulty, (2) reinforcing it on multiple occasions, (3) demonstrating its relevance to real-life problem-based scenarios, and (4) increasing student interest by showcasing its application across various settings [21, 22].

Experiential learning opportunities were introduced earlier in the revised curriculum. For example, contrary to only participating in Pre-Employment Clinical Training (PECT) in Year 4 previously, students are now attached to outpatient polyclinics and exposed to interdisciplinary teamworking with other healthcare professionals in Year 2. Students were also tasked to write a short critical reflection after each PECT activity to gain meaningful insights into the social determinants of health and into how pharmacists have the potential to take charge and act against health inequity [23]. These were done concurrently during the semester with classes to connect theoretical content with its application in clinical practice.

### Data collection

A qualitative, pre-post study design was conducted on the Class of 2024. A mixed-methods approach was conducted using two types of instruments – questionnaires and interviews.

Recruitment of NUS Pharmacy students for the interviews was conducted by a research assistant who has no responsibility in the design and teaching of the pharmacy curriculum. During recruitment in AY20/21 and AY21/22 respectively, an invitation email was sent out to the students to obtain voluntary informed consent to participate in the questionnaires and semi-structured interviews. Students who consented were then provided with the attached participant information sheet and consent form.

**Fig. 2:**
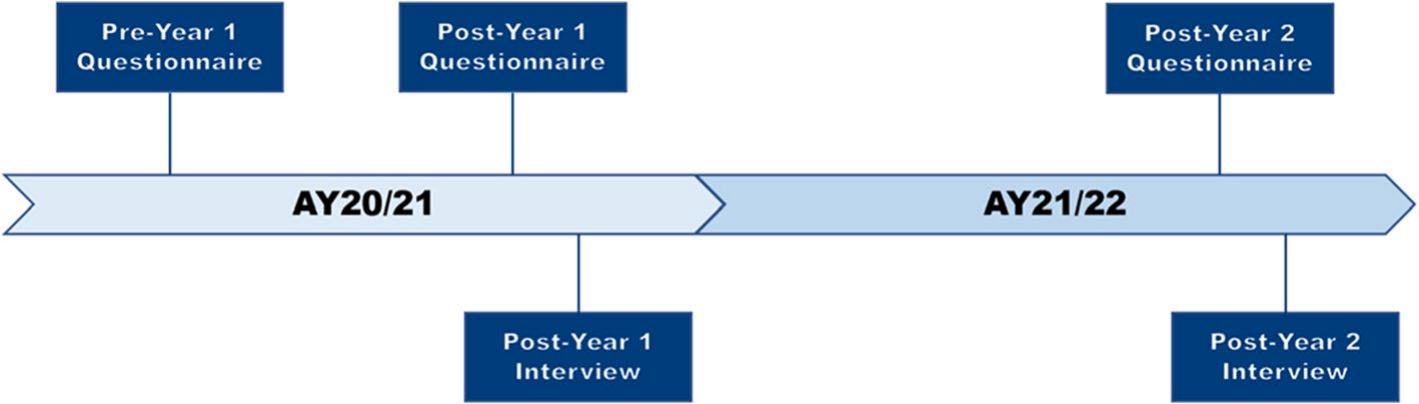
Instruments in the pre-post study design

Voluntary open-ended questionnaires were disseminated across three timepoints in AY20/21 and AY21/22 (Fig. 2), while voluntary semi-structured interviews were conducted at the end of each Academic Year. The interviews explored specific topics in-depth [24, 25], and aimed to understand students’ experiences and perceptions to rationalise the results obtained from the questionnaires [26].

Pilot testing was not conducted for both data collection methods as the instruments utilised in the study were unambiguous and straightforward in nature. Multiple internal reviews were done to enhance the validity and clarity of the instruments. Furthermore, the participants were also assumed to be highly familiar with the topic of interest since they were directly enrolled in the new spiral integrated curriculum, hence the questions involved in the instruments would be easy for the participants to comprehend and respond to accordingly.

#### Data collection in AY20/21 – Year 1

The Pre-Year 1 and Post-Year 1 questionnaires were disseminated during AY20/21 Semester 1 before the start of the Academic Year and after their final examinations in Semester 2 respectively. The same questionnaire was used for both tests, posing two questions—*one asking what health advocacy means to the students, and one asking them to cite an example of how pharmacists can contribute to health advocacy.* The Post-Year 1 interviews consisted of 11 open-ended guiding questions. Both questionnaire and interview guiding questions can be found in Additional file 1: Appendix 2.

#### Data collection in AY21/22 – Year 2

At the end of AY21/22 Semester 2, the same cohort of students was invited to participate in another questionnaire. Considering the decrease in response rate from the Pre-Year 1 to Post-Year 1 questionnaire, which could be attributed to students’ lack of inclination to participate in school-related activities after examinations, the Post-Year 2 questionnaire was disseminated before their final examinations to increase response rate. This would reduce the risk of non-response bias and preserve data quality.

A different set of questions was used in the Post-Year 2 questionnaire to avoid repetition and thus survey fatigue. These questions aimed to elicit what students thought a pharmacist can do in common, day-to-day scenarios. *Question 1 was set in a patient interaction scenario, while Questions 2 and 3 discussed reactions to broader healthcare news on different media platforms.* The different question settings provided ample opportunity for students to articulate their thoughts, thereby eliciting the depth of their internalisation of health advocacy.

Similar to the Post-Year 1 interviews, the Post-Year 2 interviews also aimed to determine changes in students’ level of internalisation of health advocacy after undergoing the integrated curriculum, and to recognise their driving factors, if any. The Post-Year 2 interviews consisted of 6 guiding questions. Both questionnaire and interview guiding questions can be found in Additional file 1: Appendix 2.

### Evaluation framework for coding questionnaire responses

Based on the concept of critical reasoning from generic questioning [27], if health advocacy was internalised and formed the basis for action, students would be able to incorporate its concepts in their responses. As there are no standardised methods for measurements of critical reasoning [28], the model proposed by Westheimer and Kahne [29] was adapted as a means to evaluate students’ degree of internalisation of health advocacy.

Westheimer and Kahne’s Good Citizenship Model identified three conceptions of a “good citizen” – “Personally Responsible Citizen”, “Participatory Citizen”, and “Justice-Oriented Citizen”. A “Personally Responsible Citizen” would uphold an individualistic sense of responsibility in maintaining oneself as a good citizen. Meanwhile, a “Participatory Citizen” would surpass an individualistic viewpoint and consciously organise and lead community efforts to solve social problems. Superlatively, a “Justice-Oriented Citizen” would critically assess and attempt to solve the social, political and economic root causes of inequity.

These categories were formed by the efforts of various teams of educators and reflected ideals that aligned with the educational aims of practitioners, including educators and curriculum designers [29]. The distinct framing in this educational model renders it a useful analytical tool when adapted in the context of health advocacy to highlight differences in the internalisation of health advocacy.

While the Good Citizenship Model required citizens to be of good character intrinsically to be categorised minimally, not all questionnaire responses assessed in this study displayed a sufficient individualistic responsibility of health advocacy to qualify for the minimal level. Such responses should neither be disregarded during the categorisation process to avoid cherry-picking of responses which would introduce bias, nor should they be qualified as “Personally Responsible” as they are misaligned with its categorical definition and would threaten the validity of the model if they were to be categorised as such. To account for these responses, an additional level of categorisation was conceived – the “Understanding Pharmacist” level.

Questionnaire responses were assessed and categorised into the four levels (Table 1) – Level 1 ‘Understanding Pharmacist’, Level 2 ‘Personally Responsible Pharmacist’, Level 3 ‘Participatory Pharmacist’, and Level 4 ‘Justice-Oriented Pharmacist’. All questionnaire responses were coded using a computer-assisted qualitative data analysis software, NVivo.

**Table 1:** Evaluation framework for assessing the level of internalisation of health advocacy

Level of internalisation	Description	Core assumptions	Sample action
Level 1: Understanding Pharmacist	Comprehends the concept of health advocacy and its importance	Informed of pharmacists’ role in advocating for health as healthcare providers	Is aware of the need to promote healthy living
Level 2: Personally Responsible Pharmacist	Advocates for the individual	Acts as a socially responsible healthcare provider and educates patients through counselling and advising	Educates patients on lifestyle modifications, attempts to understand patients’ beliefs and addresses patients’ health-related misconceptions
Level 3: Participatory Pharmacist	Advocates for the community	Actively participates and/or initiates programmes for the community	Organises nationwide campaigns to promote healthy living
Level 4: Justice-Oriented Pharmacist	Ideates innovative strategies to solve public health issues on a societal level by addressing root causes	Critically evaluates social, economic, and political systems to identify and address underlying causes of health inequity by initiating system-wide changes	Understands the barriers to cultivating healthy living habits and overcomes them by implementing healthy eating policies in schools and workplaces

### Interview procedures

Each interview began with general, context-setting questions to initiate conversation about the interviewee’s beliefs. Subsequently, specific core questions revealed how the curriculum led to an improvement in students’ internalisation of health advocacy, or lack thereof [26]. Given the flexibility of semi-structured interviews, follow-up questions could be asked to probe further [24, 30].

All students were interviewed via ZOOM video conferencing, which provided the flexibility to overcome resistance to participate in in-person interviews due to inconvenience and COVID-19 restrictions. Individual interviews rather than focus groups were conducted to understand individual behaviour, experiences, and opinions.

To consolidate the interview responses and identify recurring themes, a thematic analysis was conducted [31] and coded using NVivo.

### Data analysis and evaluation process

During the analysis and evaluation process for both questionnaire responses and interview transcripts, the researchers methodically adhered to a structured workflow. The researchers preliminarily familiarised themselves with the questionnaire responses and interview transcripts content. Next, the two researchers coded the data independently. Each researcher categorised the questionnaire responses according to the evaluation framework in Table 1, and conducted the thematic analysis of the interview transcripts by identifying and categorising recurring and prominent themes. The Pre-Year 1 questionnaire, Post-Year 1 questionnaire, and Post-Year 2 questionnaire were treated as separate datasets, and data analysis was conducted distinctly for each dataset. The results were then evaluated together to allow for comparison between the different datasets. Similarly, the Post-Year 1 interview and Post-Year 2 interview were coded distinctly, and a separate thematic analysis was conducted for each dataset before comparing and evaluating the results and trends between both datasets.

Both researchers were final year university students enrolled in NUS Pharmacy, but were completely uninvolved in the new spiral integrated curriculum. Their academic background endowed them with a requisite level of familiarity with the subject matter, including the scientific and technical jargon frequently encountered in the students' questionnaire and interview responses. This unique perspective facilitated the coding process while mitigating potential biases as the researchers approached the analysis objectively due to their lack of direct involvement in the new curriculum under investigation.

### Ethical considerations

For all questionnaires and interviews, participants were not approached directly by the researchers and faculty during recruitment. Open invitation to participate was made by a research assistant uninvolved in teaching the pharmacy curriculum. It was emphasised that participation was voluntary and failure to participate would not result in any adverse consequences, nor was it part of any academic assessment. No personal information was requested nor needed. Recorded interview audio clips were transcribed and destroyed no later than two weeks once transcripts were verified as accurate.

All personal data unintentionally revealed in the interview were masked or coded in the transcripts and all research results were de-identified. Responses were coded by two separate researchers and any disagreements were adjudicated by the Principal Investigator.

Approval for the research protocol was granted by the Learning and Analytics Committee on Ethics (LACE) of NUS.

## Results

### Analysis of coded questionnaire responses

Responses to each question were coded as individual responses. In AY20/21, within the Class of 2024 cohort of 150 students, a total of 215 (72%) responses were obtained for the Pre-Year 1 questionnaire (2 questions), and 126 (42%) responses were obtained for the Post-Year 1 questionnaire. In AY21/22, 383 (85%) responses were obtained for the Post-Year 2 questionnaire (3 questions).

The Mann–Whitney U test was used to compare the distribution of levels across responses between the Pre-Year 1 and Post-Year 1 questionnaires, and between the Pre-Year 1 and Post-Year 2 questionnaires. Results were presented as median (IQR) (Table 2). All comparisons were one-tailed with a significance level of 0.05.

**Table 2:** Statistical analysis of questionnaire results

Level of internalisation	Pre-Year 1 Questionnaire	Post-Year 1 Questionnaire	Post-Year 2 Questionnaire	Mann–Whitney U test comparing Pre-Year 1 and Post-Year 1 Questionnaires	Mann–Whitney U test comparing Pre-Year 1 and Post-Year 2 Questionnaires
Level 1: Understanding Pharmacist	16 (7.4%)	7 (5.5%)	9 (2.3%)		
Level 2: Personally Responsible Pharmacist	192 (89.3%)	116 (92.1%)	286 (74.7%)		
Level 3: Participatory Pharmacist	6 (2.8%)	2 (1.6%)	59 (15.4%)		
Level 4: Justice-Oriented Pharmacist	1 (0.5%)	1 (0.8%)	29 (7.6%)		
Total number of completed answers	215	126	383		
Median level (IQR)	2 (2–2)	2 (2–2)	2 (2–2)		
*P*-value				0.44	< 0.01

There was no statistically significant difference between the median levels of health advocacy internalisation (IQR) of the Pre-Year 1 questionnaire and the Post-Year 1 questionnaire [2.0 (2.0–2.0) vs 2.0 (2.0–2.0), *p* = 0. 44]. In contrast, there was a statistically significant difference between the median levels of health advocacy internalisation (IQR) of the Post-Year 1 questionnaire and the Post-Year 2 questionnaire [2.0 (2.0–2.0) vs 2.0 (2.0–2.0), *p* < 0.01].

### Thematic analysis of Post-Year 1 interviews

Five interviewees participated in the Post-Year 1 interviews, and a thematic analysis conducted revealed their understanding of health advocacy and views on the Year 1 curriculum (Table 3).

**Table 3:** Thematic analysis of Post-Year 1 interviews

Themes and Description	Sample interview excerpts
**Understand the need for strong pharmacological knowledge in health advocacy**	
The interviewees commonly believed that extensive pharmacological knowledge is important for health advocates. They recognised that pharmacists’ responsibilities include dispensing and counselling on drug use and lifestyle changes.	* “I think pharmacists naturally do play a role in actually promoting to the public about [the] upkeeping of health… our role is like medication experts…”* Interviewee 2 *“So what roles [do] pharmacists play… I think the first important thing for health advocacy is basically the appropriate use of medications. Teach [patients] or educate them on how to appropriately use that medication.”* Interviewee 5
**Apprehension towards pharmacists’ roles in improving health beyond the individual level towards the community level**	
The interviewees voiced reservations about their abilities to contribute to health advocacy on a macro level. Majority did not recognise pharmacists’ role in managing upstream social determinants of health as they felt that pharmacists had limited influence on the community and systems levels. This plausibly led to their belief that pharmacists’ role in health advocacy is limited to the individual level in direct patient care settings. However, one interviewee acknowledged that the curriculum was effective in empowering students to advocate for health beyond the individual level.	*“I think my role next time will be more towards maintaining health, not so much on prevention. And I think the role is not very big. In the sense that I think, perhaps advertisements online have a larger reach and impact than what pharmacists are telling the patient…”* Interviewee 3 “Some of us may feel that pharmacist[s] may not play such an important role in the healthcare setting, especially in health advocacy. So, with this new curriculum rolled out, in Year 1, you are already introduced [to] how important [your role as a pharmacist is] and what kind of role you play, and then we can build on that in the future.” Interviewee 2
**Desire for more experiential learning opportunities**	
The interviewees found the idea of experiential learning beneficial and showed interest in undertaking more opportunities to apply theoretical knowledge into practice to enhance their understanding of key concepts and skills.	*“I think that [experiential learning] helps quite a lot because they [put] our skills to actual use, like going out [to] the field and talking to actual patients.”* *Interviewee 1* *“If we are able to do [virtual befriending sessions with] more people in the future, I think that's quite inspiring for me, that's why I think it really excites me to be learning more about health advocacy and actually doing more of these—getting more experience.”* *Interviewee 2* *“[With regards to how the curriculum can be further improved] More community visits… Maybe if things were in real-life [in contrast to virtually during the COVID-19 pandemic], maybe that will be a better learning experience for all students.”* *Interviewee 4*
**Varying opinions on the incorporation of health advocacy concepts in the curriculum**	
The interviewees expressed conflicting sentiments about the curriculum’s effectiveness in teaching health advocacy. Some desired health advocacy concepts to be taught more explicitly. As internalising health advocacy does not occur intuitively for students, it should be fostered through clearer emphasis in the curriculum.	* “Maybe if [the professors] explicitly highlight [health advocacy] first, and then, tell us what is the importance of health advocacy… [and] cover it like an actual lesson itself so that we have better awareness of health advocacy.”* Interviewee 1 *“[The curriculum] can have more case studies in terms of real-life examples on how pharmacists may be able to contribute to health advocacy.”* Interviewee 5
Contrarily, some interviewees felt that the curriculum was already effective in inculcating health advocacy and displayed enhanced awareness of it.	*“From what we've covered in Year 1, I think they're trying to impress on us that it's not just about providing healthcare but also managing health, just to prevent people from even being in the state to need healthcare services.”* Interviewee 2 “*If you make the drug cheaper here in a way, then you tend to promote health because patients are able to purchase this drug and utilise it.”* Interviewee 4

Generally, internalisation of health advocacy was lacking among most interviewees, and many felt that curricular integration was not apparent enough. In consideration of this, more was done in the Year 2 curriculum to make the integration between basic, clinical and system sciences clearer, such as making more references to previous learning materials to help students interlink different topics. Furthermore, acknowledging students’ enthusiastic feedback on experiential learning, more opportunities were also incorporated in Year 2 for them to integrate content and skills and translate them into practice.

### Thematic analysis of Post-Year 2 interviews

Six students were interviewed after undergoing the Year 2 pharmacy curriculum, and another thematic analysis was conducted (Table 4).

**Table 4:** Thematic analysis of Post-Year 2 interviews

Themes and Description	Sample interview excerpts
**Integration between basic, clinical, and systems sciences facilitated the internalisation of health advocacy concepts in students**	
The interviewees could clearly connect basic, clinical and systems sciences and apply them in pharmacy practice, as a result of curriculum integration. They displayed holistic understanding of the local healthcare landscape, and the social, political, economic, and cultural factors that affect health. They also acknowledged that health advocacy is an intrinsic responsibility of pharmacists. Ideally, this would transpire them to practise health advocacy on an individual, population, and ultimately, systems level. For instance, one interviewee suggested using online platforms to reach out to a wider audience beyond individual patients.	*“We can see the integration… in the skills module [where] we use the information and content that we learnt in the systems modules. And there is an overarching module that teaches us law regulations, ethics, and related matters.”* Interviewee 10 *“We don’t view [health advocacy] as a separate part of being a pharmacist.”* Interviewee 6 *“[There are modules that cover] social issues regarding inequality, pharmacoeconomics, pharm[acy] law… these develop us holistically as pharmacists, other than just the [basic and clinical] sciences, like how we treat patients, choice of therapy, et cetera…”* *Interviewee 8* *“[Health advocacy can be practised on both an individual and community level.] For example, during dispensing, that is a personal level. And maybe, using the public sites like the government websites [can help us] to expand to the public as well, so we can reach out to more people, and maybe tell them what does this medicine do, and what are the possible side effects and what they should look out for.”* Interviewee 7
**Increasing complexity of content taught**	
The interviewees recognised how previous concepts were reinforced in increasing intricacy, a cornerstone of a spiral curriculum.	*“How [the professors] introduced to us, section by section, interacting with patients. So in Year 1 Semester 1, we [learnt] only history taking. In [Year 2 Semester 1], [we learnt about] diagnosing minor ailments. This semester, we [learnt about the] suggest[ed] treatments for minor ailments. So that step-by-step gradual exposure has also helped me to learn at a better rate.”* Interviewee 6 *“[The curriculum] is organised like a ladder. The foundation is built in Year 1… there are then different systems modules coming in. And even on these systems modules, there are more systems modules that are built on it.”* Interviewee 8
**Roleplaying and experiential learning helped students put knowledge into clinical or community care perspectives**	
The interviewees viewed roleplaying in patient interaction simulations as ideal avenues to apply their knowledge. The interviewees also appreciated experiential learning and many cited PECT particularly, where students are attached to training institutions including community pharmacists, polyclinics, and hospitals.	*“Knowledge [from the patient communication and skills module] is integrated into communicating information to patients or trying to get some information from patients… so we can suggest the appropriate pharamcological management, non-pharmacological management and monitoring and follow-up, et cetera, in the pharmaceutical care plan.”* Interviewee 11 *“I expect to do more experiential learning to actually try out the skills and [use the] content that we learnt, so we can actually apply it, instead of simply just learning about it.”* Interviewee 10 *“[I would expect to experience] more PECT rotations and more time out of the [classroom] context and appreciating what pharmacists do. I think that, to me, plays the biggest role in helping to shape what I view about pharmacists and how pharmacists can make a huge impact in this healthcare system and continue to educate the general public.”* Interviewee 9
**Curriculum instilled greater confidence in students to practise professionally**	
The interviewees expressed greater confidence towards their professional practice by virtue of the knowledge, attitude and experience gained from the curriculum.	*“I am better able to make judgements in the future as a future healthcare provider.”* Interviewee 9 *“I can help my family [by practising] health advocacy with their health… with all these integrated knowledge that I have learn from the curriculum.”* Interviewee 11

## Discussion

The insignificant improvement in students’ level of internalisation of health advocacy after Year 1 can be attributed to the lack of emphasis on health advocacy. During the Post-Year 1 interviews, many suggested that clearer definitions and objectives could be set for health advocacy (Table 3). The implicit nature of health advocacy is a potential barrier in improving its internalisation as students do not perceive it to be as important as clinical and basic sciences [32]. Furthermore, students still regarded it as an isolated concept, failing to establish its connection with basic, clinical, and systems sciences. Generally, while health advocacy could be made more apparent, it should be woven into the curriculum longitudinally to encourage students to formulate its practice into habit [33, 34].

Moreover, many students believed that pharmacists have limited roles in advocating for health beyond individual patient care (Table 3). This is possibly due to the Year 1 curriculum being the beginning of the spiral curriculum, where concepts were taught on an introductory level and more emphasis was placed on building strong conceptual foundations. For instance, the Year 1 curriculum introduced the foundational concepts of healthcare financing and rising healthcare costs (Fig. 1). While these would help students understand the economic backdrop of healthcare, students would have yet to be exposed to how these concepts can be employed on a population and systems level, since these would be taught in subsequent years of the curriculum in increasing complexity. Therefore, the Post-Year 1 findings on the lack of internalisation of health advocacy in students are not necessarily indicative of curricular flaws, and instead, may be an inevitable attribution of the rudimentary yet quintessential beginning point of the spiral curriculum.

Following the Year 2 curriculum, there was an improvement in the internalisation of health advocacy. In line with the spiral curriculum, the Year 2 curriculum reinforced previous concepts and built more complex ideas on them (Table 4). Moreover, integration enabled students to see interrelationships more clearly, improved their retention of information by linking health advocacy concepts taught in Year 1 to specific contexts [35], and subsequently, facilitated their application of such knowledge in actual settings [36]. This may explain the shift from integration being perceived as lacking in the Year 1 curriculum to more apparent in the Year 2 curriculum as reflected in the thematic analysis.

The Post-Year 2 interviews suggested that roleplaying and experiential learning are beneficial in teaching health advocacy (Table 4). They expose students to patients’ multifaceted ideas, concerns, and expectations, humanising patients and spurring students to consider patients’ attitudes with their medical conditions [37]. Moreover, roleplaying and experiential learning are intuitive platforms for students to consolidate information learnt and apply them to real-world scenarios [38, 39]. The Year 2 curriculum introduced these experiential learning opportunities, such as PECT (Table 4). The active community involvement provides an authentic and emotional lens for students to appreciate the impacts of social organisation, socioeconomic factors, health policies, and other factors on patient decisions and health outcomes [23, 40]. Hence, through meaningful reflection on their experiences, students would be more empowered and confident in their abilities to tackle underlying upstream factors of health inequity during their undergraduate journeys and during their professional careers [41–43].

The overall improvement in students’ internalisation of health advocacy reflects the effectiveness of spiral integrated curricular designs in instilling health advocacy. This is consistent with cognitive psychology theories that have found that interweaving the teaching of basic sciences with that of clinical sciences sets the context for the knowledge learnt in the formal academic setting [44]. As opposed to the traditional block curriculum, where basic sciences concepts are taught in isolation, the integrated curriculum enables students to organise and connect the fundamental concepts to clinical practice more easily [36, 45]. This also improves the retention of knowledge as it provides opportunities for students to apply their knowledge within a reasonable timeframe [22, 35, 46].

### Study limitations

This study has several potential limitations.

Firstly, open-ended questions in the questionnaires relied on students’ abilities to give articulate accounts of their opinions, which might not accurately reflect their underlying attitudes towards health advocacy. Furthermore, the coding process might be subjective as it depended on the researchers’ interpretation of students’ responses. To overcome bias in the assessment process, two researchers independently coded the questionnaire responses and themes recurring in the interview responses. All differences in opinions were adjudicated by the Principal Investigator.

Secondly, interviews were conducted on a voluntary basis, potentially introducing voluntary response bias. There was likely an oversampling of students who were more opinionated about health advocacy and the curriculum, and thus, an overstatement of the effects of the curriculum on the internalisation of health advocacy.

Thirdly, the limited sample size obtained for the interviews and any potential bias introduced as a result of the voluntary nature of the interview recruitment process contributed to a lack of diversity in the data, thus making it difficult to ascertain if data saturation has been reached.

It is acknowledged that the quantitative and qualitative methods used each have their own limitations and biases [47], and their impact on the internal validity of this study may have been accentuated by the lack of a control group. To enhance the validity of the study findings, a mixed methods research design was used. Since the results of the questionnaires and interviews converged and corroborated with each other, the inherent biases of each method were counteracted, thus improving the credibility of the findings in this study [48, 49]. Additionally, the mixed methods research design offered multiple perspectives on the integrated curriculum’s impact on the internalisation of health advocacy, thus producing a comprehensive and holistic understanding of the obtained results [50, 51].

### Future directions

While there is a lack of evaluation on whether the participants would go on to actively practise health advocacy during their professional careers, further follow-up evaluative studies can be conducted in the long run on the same cohort of students to investigate this. It is hoped that future studies can identify other crucial elements and areas of improvement in this integrated curriculum, and potentially, guide the implementation of similar integrated curricula in postgraduate pharmacy education, other pharmacy and even other healthcare disciplines educational institutions.

Moreover, taking into account that the experiential learning opportunities in this integrated curriculum are primarily in patient care settings, designers of similar prospective curricula may also partner with social and political institutions to expose students to grassroots organisation and policy making [33]. This potentially enables students to internalise the scope of their roles in health advocacy more comprehensively beyond patient and community care settings.

## Conclusion

This paper discussed and evaluated the effectiveness of a spiral integrated undergraduate Pharmacy curriculum on the internalisation of health advocacy in pharmacy students. The improvement in internalisation of health advocacy in the study population suggests promising results of spiral integrated curricular designs which integrate basic, clinical, and system sciences.

### Supplementary Information

**Additional file 1:** **Appendix 1. **CanMEDS role: health advocate. **Appendix 2. **Questionnaire questions.

**Additional file 2. **

## Data Availability

All data generated or analysed during this study are included in the supplementary information files of this article.

## References

[1] Low LL, Wah W, Ng MJ, Tan SY, Liu N, Lee KH (2016). Housing as a social determinant of health in Singapore and Its association with readmission risk and increased utilization of hospital services. Front Public Health.

[2] Hertzman C, Frank J, Evans R. Heterogeneities in health status and the determinants of population health. In Evans RG, Barer ML, Marmor TR. (Eds.), Why are some people healthy and others not? The determinants of health populations (1st ed.). Routledge; 1994. p. 67–92. 10.4324/9781315135755.

[3] Gehlert S, Sohmer D, Sacks T, Mininger C, McClintock M, Olopade O (2008). Targeting health disparities: a model linking upstream determinants to downstream interventions. Health Aff.

[4] Kellar J, Deal H, Fitz Patrick B, et al. AFPC educational outcomes 2017. Ottawa: Association of Faculties of Pharmacy of Canada; 2017.

[5] Hubinette M, Dobson S, Scott I, Sherbino J (2017). Health advocacy. Med Teach.

[6] Royal College of Physicians and Surgeons of Canada. Health advocate. CanMEDS Role: Health Advocate: The Royal College of Physicians and Surgeons of Canada. 2015. Retrieved Oct 19, 2022, from https://www.royalcollege.ca/rcsite/canmeds/framework/canmeds-role-health-advocate-e.

[7] Pauling EE, Nguyen TT, Valentino AS, Ducker Coleman M (2022). Using the Pharmacists’ Patient Care Process to address social determinants of health in patients with diabetes. J Am Pharm Assoc.

[8] Williams DR, Costa MV, Odunlami AO, Mohammed SA (2008). Moving upstream: how interventions that address the social determinants of health can improve health and reduce disparities. J Public Health Manag Pract.

[9] Ivory K, Bandler L, Hawke C, Armstrong B (2013). A clinical approach to population medicine. Clin Teach.

[10] Quirk ME, Harden RM. Curriculum planning and development. In: Dent JA, Harden RM, Hunt D, Hodges BD, editors. A practical guide for medical teachers. 5th ed. essay, Elsevier; 2017. p. 4–12.

[11] Gonzalo JD, Haidet P, Papp KK, Wolpaw DR, Moser E, Wittenstein RD, Wolpaw T (2017). Educating for the 21st-century health care system: an interdependent framework of basic, clinical, and systems sciences. Acad Med.

[12] Harden RM (2000). The integration ladder: a tool for curriculum planning and evaluation. Med Educ.

[13] Pearson ML, Hubball HT (2012). Curricular integration in pharmacy education. Am J Pharm Educ.

[14] Barzak MY, Ball PA, Ledger R (2001). The rationale and efficacy of problem-based learning and computer assisted learning in pharmaceutical education. Pharm Educ.

[15] Schwartz A, Elstein A (2006). Clinical reasoning in medicine.

[16] Armbruster AL, Henson BN, Alsharif NZ (2022). A call to action for a programmatic approach to addressing health disparities and cultural competency in pharmacy education. Am J Pharm Educ.

[17] Hsia SL, Landsfeld A, Lam K, Tuan RL (2021). Implementation and evaluation of a 10-week health equity curriculum for pharmacy students. Am J Pharm Educ.

[18] Keller LO, Strohschein S, Lia-Hoagberg B, Schaffer MA (2004). Population-based public health interventions: practice-based and evidence-supported. Part I Public Health Nursing.

[19] Koster A, Schalekamp T, Meijerman I (2017). Implementation of Competency-Based Pharmacy Education (CBPE). Pharmacy (Basel).

[20] Bruner J (1960). The Process of Education.

[21] Harden RM (1999). What is a spiral curriculum?. Med Teach.

[22] Husband AK, Todd A, Fulton J (2014). Integrating science and practice in pharmacy curricula. Am J Pharm Educ.

[23] Dharamsi S, Richards M, Louie D, Murray D, Berland A, Whitfield M, Scott I (2010). Enhancing medical students’ conceptions of the CanMEDS health advocate role through international service-learning and critical reflection: a phenomenological study. Med Teach.

[24] Liamputtong P. Handbook of research methods in health social sciences. Springer Singapore; 2019. 10.1007/978-981-10-5251-4.

[25] Silverman D (2017). Doing qualitative research.

[26] DeJonckheere M, Vaughn LM (2019). Semistructured interviewing in primary care research: a balance of relationship and rigour. Family Medicine and Community Health.

[27] Sykes AH, Azfar J (2019). Constructing understanding through critical questioning: a comparative study of first year undergraduate and postgraduate writing and communication classes. Asian J Scholarship Teaching Learning.

[28] Moore T (2004). The critical thinking debate: How general are general thinking skills?. High Educ Res Dev.

[29] Westheimer, J., & Kahne, J. What kind of citizen? Political choices and educational goals. Encount Theory History Educ. 2008;4. 10.24908/eoe-ese-rse.v4i0.658.

[30] Bryman A (2016). Social Research Methods.

[31] Braun V, Clarke V (2006). Using thematic analysis in psychology. Qual Res Psychol.

[32] Douglas A, Mak D, Bulsara C, Macey D, Samarawickrema I (2018). The teaching and learning of health advocacy in an Australian medical school. Int J Med Educ.

[33] McDonald M, Lavelle C, Wen M, Sherbino J, Hulme J (2019). The state of health advocacy training in postgraduate medical education: a scoping review. Med Educ.

[34] Towle A, Godolphin W, Hubinette M, Macdonald S, Hewitt C (2013). The CanMEDS role of health advocate in postgraduate education at UBC: summary report.

[35] Rosse C (1974). Integrated versus discipline-oriented instruction in medical education. Acad Med.

[36] Lam TP, Irwin M, Chow LWC, Chan P (2002). Early introduction of clinical skills teaching in a medical curriculum – factors affecting students’ learning. Med Educ.

[37] Greenhalgh T, Hurwitz B (1999). Narrative based medicine: why study narrative?. BMJ.

[38] Murphy S. Stories are better than lectures at teaching us about health. The Conversation. 2017. Retrieved September 22, 2022, from https://theconversation.com/stories-are-better-than-lectures-at-teaching-us-about-health-71682.

[39] Rodrigo C, Tedla N, Thomas S, Polly P, Herbert C, Velan G, Saunders DN (2019). Using narratives to teach students enrolled in science and medical science bachelor's degree programs. Med Sci Educ.

[40] Fung OW, Ying Y. Twelve tips to center social accountability in undergraduate medical education. Med Teach. 2021:1–7. 10.1080/0142159X.2021.1948983. 10.1080/0142159X.2021.1948983 34294021

[41] Boroumand S, Stein MJ, Jay M, Shen JW, Hirsh M, Dharamsi S (2020). Addressing the health advocate role in medical education. BMC Med Educ.

[42] Cantor JA (1995). Experiential learning in higher education: linking classroom and community.

[43] Hunt JB, Bonham C, Jones L (2011). Understanding the goals of service learning and community-based medical education: a systematic review. Acad Med.

[44] Hsia SL, Gruenberg K, Nguyen J, La A, MacDougall C (2023). Student performance outcomes and perceptions in two content areas in conventional versus integrated pharmacy curricula. Am J Pharm Educ.

[45] Schmidt HG (1983). Problem-based learning: rationale and description. Med Educ.

[46] Brauer DG, Ferguson KJ (2015). The integrated curriculum in medical education: AMEE Guide No. 96. Med Teach.

[47] Harris LR, Brown GTL (2010). Mixing interview and questionnaire methods: Practical problems in aligning data. Pract Assess Res Evaluation.

[48] Greene JC, Caracelli VJ, Graham WF (1989). Toward a conceptual framework for mixed-method evaluation designs. Educ Eval Policy Anal.

[49] Greene J, McClintock C (1985). Triangulation in evaluation: design and analysis issues. Eval Rev.

[50] Andrew S, Halcomb EJ, Borbasi S, Jackson D (2012). Mixed method research. Navigating the Maze of Research: enhancing nursing & midwifery practice.

[51] Simons L, Lathlean J, Gerrish K, Lacey A (2010). Mixed Methods. The research process in nursing.

