# Supplementary material for: Evaluating the internalisation of the intrinsic role of health advocacy of student pharmacists in a new integrated Bachelor of Pharmacy curriculum: a mixed-methods study
Source: BMC Med Educ. 2023 Nov 27;23:900. doi: 10.1186/s12909-023-04877-y (PMC10680209; doi:10.1186/s12909-023-04877-y)
Supplement: Supplementary file 1 — Additional file 1: Appendix 1. CanMEDS role: health advocate. Appendix 2. Questionnaire questions. [file 12909_2023_4877_MOESM1_ESM.docx]

**Appendix 1**

CanMEDS Role: Health Advocate

| **Key competencies**  Physicians are able to: | **Enabling competencies** |
| --- | --- |
| 1. Respond to an individual patient’s health needs by advocating with the patient within and beyond the clinical environment | 1.1 Work with patients to address determinants of health that affect them and their access to needed health services or resources  1.2 Work with patients and their families to increase opportunities to adopt healthy behaviours  1.3 Incorporate disease prevention, health promotion, and health surveillance into interactions with individual patients |
| 1. Respond to the needs of the communities or populations they serve by advocating with them for system-level change in a socially accountable manner | 2.1 Work with a community or population to identify the determinants of health that affect them  2.2 Improve clinical practice by applying a process of continuous quality improvement to disease prevention, health promotion, and health surveillance activities  2.3 Contribute to a process to improve health in the community or population they serve |

**Appendix 2**

Questionnaire Questions

**Pre-Year 1 and Post-Year 1 Questionnaires**

1. In a single sentence, describe what health advocacy means to you.
2. In a single sentence, suggest an example of how a pharmacist can contribute to health advocacy in Singapore.

**Post-Year 2 Questionnaire**

1. A 35-year-old female patient is referred to a pharmacist-run diabetes clinic by her primary care physician for her newly diagnosed Type 2. She attends the clinic appointment with her husband. The patient confesses to the pharmacist that although she was surprised to be diagnosed with diabetes at a young age, she believes her body still needs all the sugar it can get because she is still young and planning to get pregnant. Her husband nods in agreement with her sentiment.

Question: What would the pharmacist do about such notions?

1. <https://www.straitstimes.com/singapore/health/people-in-singapore-less-healthy-and-covid-19-may-worsen-situation-national>

After reading this headline of this news article, what do you think a healthcare provider would do about the current situation?

1. https://www.youtube.com/watch?v=n-rKc44Q4UA

Title: 15% of Singaporeans will be obese in 5 years’ time if left unchecked: NHG report (50 seconds)

After watching the video, what actions would a member of the healthcare workforce take, with regards with this issue?

Semi-Structured Interview Guide

**Post-Year 1 Interviews**

Determining the understanding of health advocacy: Elaboration on 2 questions asked in the survey:

1. What role(s) do you think Pharmacists have to play as health advocates in Singapore society? Give some examples of how pharmacists can be advocates for good health at the individual or population level.
2. How might you see yourself as an advocate of good health in future as a pharmaceutical and/or healthcare professional?
3. What three characteristics best describe an effective health advocate to you?
4. Do you feel that you have a basic grasp of what “health advocacy” entails, and ready to move on to the next phrase? Why or why not?

Determining the factors which drive this change (or not) regarding the level of understanding.

1. Has your understanding of “health advocacy” changed after your first year as a pharmacy undergraduate? If yes, to what extent has it changed?
2. To what factors would you attribute this change in understanding of “health advocacy” over the academic year? (Consider your experiences with the curriculum/modules, teaching staff, co-curricular activities, enrichment programmes etc.) If your understanding of “health advocacy” has not changed or changed only minimally, how were the above factors (curriculum/modules, teaching staff, co-curricular activities, enrichment programmes etc.) aligned with your initial understanding of “health advocacy”?
3. In general, what elements of teaching and learning in the pharmacy curriculum e.g. design of modules, projects, teaching modes, assessment, teaching staff, learning environments (physical and virtual) would you think have an influence on the promotion of health advocacy among Pharmacists? How might these elements of teaching and learning be used to deepen the understanding of “health advocacy” among Pharmacists?
4. What elements of the co-curriculum offered by the Department of Pharmacy eg. Co-curricular or enrichment programmes etc. would you think have an influence on the promotion of health advocacy among Pharmacists? How might these elements of the co-curriculum be used to deepen the understanding of “health advocacy” among Pharmacists?
5. As the new pharmacy curriculum is very much based on basic, clinical and system sciences integration, was this integration apparent to you and does it contribute to your understanding of “health advocacy”?
6. How can we improve on this integration?
7. What kinds of modules, programmes and activities related to the promotion of “health advocacy” would you expect to experience in your second year? What kinds of modules, programmes and activities related to the promotion of “health advocacy” would you personally like to see or experience? Give some examples.

**Post-Year 2 Interviews**

1. Has the Year 2 curriculum further deepened your understanding of health advocacy by pharmacists beyond the Year 1 curriculum? If there is no change, why? And then if yes, why and what are the stand-out elements to the Year 2 curriculum?
2. Imagine a prospective Pharmacy student asks you to explain how the new Pharmacy program is organized. How would you explain its structure?
3. The new Pharmacy curriculum is based on the integration of basic, clinical and system sciences. Which elements of the program best highlight the integration, and was this integration apparent to you?
4. How does this integrated curriculum contribute to your understanding of health advocacy? Or, how does it not contribute to your understanding of health advocacy?
5. Looking ahead, what kinds of modules, programmes and activities related to the promotion of health advocacy would you expect to experience in your third year?
6. What kinds of modules, programmes and activities related to the promotion of health advocacy would you personally like to see or experience?
