# Supplementary material for: Evaluating the internalisation of the intrinsic role of health advocacy of student pharmacists in a new integrated Bachelor of Pharmacy curriculum: a mixed-methods study
Source: BMC Med Educ. 2023 Nov 27;23:900. doi: 10.1186/s12909-023-04877-y (PMC10680209; doi:10.1186/s12909-023-04877-y)
Supplement: Supplementary file 2 — Additional file 2. [file 12909_2023_4877_MOESM2_ESM.zip › Raw Data/Post Year 1 Interview Transcripts/Post Year 1_Interviewee 1_Transcript.docx]

# Transcript of Post-Year 1 Interview with Interviewee 1

Interviewer:

The first question is what role(s) do you think pharmacists have to play as health advocates in Singapore society? Give some examples of how pharmacists can be advocates for good health at the individual or population level.

Student:

What role ah?

Okay, so from a... Because I am a student now. So from my point of view right, pharmacists, their role is... I think it's quite important to promote health. I think… er... one of my professors told me that there are certain.... because we are not doctors right? It doesn't mean that our role is diminished, we still have like a lot of things to play. So for example, like a non-pharmacological advice or things, key tips to improve our patients’ lifestyles.

Uh, I think Dr Koh told me like, there are some advice like that.

Even though you may think that everybody knows why, some people, to them right, they still have not heard of it, so it’s always good that we impart some of these advice to them now so that we can generally like improve their quality of life.

Interviewer:

Okay, thank you so much. So can you give me some examples of how pharmacists can be advocates for good health at the individual level and the population level?

Student:

Uh, can I quote my lecture slides?

I think I learned before

Interviewer:

Uh, sure.

Student:

Uh, on the… so the population level: I think they mention something about pharmacy committee, they are supposed to monitor the standard drug list? So basically it's like, uh, make sure which drug is appropriate or like which drug is you know the best for the patient. So this is like I think it's a form of way to help do like you know, improve patients medicine use, which goes towards health advocacy, I think.

Interviewer:

And how about the individual level?

Student:

Individual level, I think just that general dispensing advisors as well as reducing polypharmacy la, so essentially just to minimize giving the patients too many drugs so that it doesn't confuse them lor.

Interviewer:

Umm I see. Okay, so the next question. How might you see yourself as an advocate of good health in the future as a pharmaceutical or health care professional?

Student:

Why is this question quite nebulous?

I don't really know what I will do down the line. I mean, uh, if I enter healthcare right? If I practice as a pharmacist, then I will definitely practice health advocacy lah, to my patients.

Student:

Yeah, but it's hard for me to foresee what I’ll do after I graduate

Interviewer:

OK, so your future hasn't been determined yet, right?

Student:

Yeah, ‘cause there's so many pathways, right?

Interviewer:

True. OK, yeah. So what three characteristics best describe an effective health advocate to you?

Student:

Uh, three characteristics ah?

I think number one would be, uh, empathy. So empathy is very important as skills.

Because you don't really even empathize with the patients right, you don't really understand, you don’t really know like what issues he or she is facing and then you cannot advocate the right health improvements.

Number 2, I believe that... I think the need to like … The attitude of improvement is also quite important. So like always improving oneself, first you have to know your content mah, you have to know what your evidence-based medicine to like know what is the best advocate, the right health medication.

Yeah then lastly... I don’t really know what’s the last point. Is it okay if I just skip?

Interviewer:

Huh, okay, so uh, you only have the two most important characteristics in mind?

Okay, it's okay. Uh, so the next one is. Do you feel that you have a basic grasp of what health advocacy entails and ready to move on to the next phase? Why or why not?

Wow, okay, uh, for how much do I understand?

I don't think I would say that I understand everything about health advocacy. I think it’s a very large field, so to speak. There are so many aspects that encompasses advocacy. And obviously I'm a student ma, I won’t know, I don’t think I even scratch the surface of what it means to advocate health, but I would definitely like to learn about what other ways that can help to advocate health for my patients. I mean, after another, ‘cause I’m at my first year ma, so everything is not set in stone. Maybe I think I’ll

look at my education and improve from there.

Interviewer:

Yeah true. Okay, so you're ready to move on to the second year, right?

Student:

Yeah.

Interviewer:

Okay, Next up, has your understanding of health advocacy changed after the first year as a pharmacy undergrad? If yes, to what extent has it changed?

Student:

Wait, are we comparing to before I enlisted in uni?

Interviewer:

Yes, yes. So yeah, so basically how your understanding of health advocacy changed after your first year as an undergrad, yeah?

Student:

Wow, okay this question is quite....

Interviewer:

Uh, so it needs some reflection.

Student:

I don't know whether it’s repolarizing. On one hand, I feel like it changes a lot, but on the other hand it didn’t change a lot. It doesn't change much.

Student:

So okay, can I say why it changed a lot first?

Interviewer:

Yeah, yeah, sure.

Student:

So it changed a lot because in the 1st place, right before I matriculated, I have no healthcare knowledge, whatsoever, I don't know anything about a human body, medicine, treatment or dispensing advisors and all these. I know nothing about these.

So once I’m in my first year, I learned a lot about, like, what the pharmacists do, what looks professional, what roles, the job as well as the importance of health advocacy.

So with that we got right, I think it's a lot, but if you're comparing to like what a pharmacist will achieve right? I don't think it's a lot, because like I said, I like I'm only in my first year, so I don't think I barely touch upon anything about health advocacy at all. I don't know the very general advice, for example dieting, exercise, you know the importance of health... All these are very general advocate slogans in that sense

Interviewer:

So your understanding about health advocacy. You've learned a lot, but to you, it's still not much yet, right?

Student:

Okay, so uh, let me quantify. For example, let's say uh a pharmacist, her advocacy skills is like 10, very good, like know a lot about health advocacy. For me, right now I’m at one.

But compared to before I matriculate right, I'm at zero, so to me from zero to one is very significant. But then one compared to 10 is insignificant. That’s what I meant.

Interviewer:

Okay, yeah, I understand. Very interesting. Okay, so the next one is to what factors would you attribute this change in understanding of health advocacy over the academic year? So you can consider your experiences with curriculum or teaching staff, CCA or enrichment programs and so on.

Student:

Uh, so the question is like to what extent these co-curricular activities help to improve my health advocacy skills?

Interviewer:

Uh, no, to what factors would you attribute this change after the first year in understanding of health advocacy over the academic year?

Student:

I think mostly is just my university syllabus. My pharmacy syllabus.

Other than that, I don't think my co-curricular activities did much, I would say.

But something really interesting is that the pharmacy module, they actually have this... I don't know whether they are called activities, but they call it a longitudinal student program. So basically they wanted us to ...so it’s like they form groups la, basically, so Medicine they take 1 student, Pharmacy they take 1 student, Nursing they take one student, etc. Then we form one group, then we visit one patient and then we will try to gather information from him or her, as well as tried to, you know, advocate health, in that sense. Basically like we are the healthcare providers.

Yeah, so I think that that helps quite a lot because it's like, uh, you know they’re putting our skills to actual use, actually, like going out in the field and talking to actual patients.

So like it's very different compared to just sit in a classroom and learning about that because you don't really get to interact with real-life patients.

And then in pharmacy, there's this very interesting one. They want us to do 3 hours of compulsory volunteering. So I have to go and do my volunteer. So one of my volunteer is just basically same thing as the longitudinal program. Basically, this is also the same one. I just find some of my allied health volunteers, then we go and visit patient and just talk to them.

So I think these two help a lot in understanding what a health advocate does, especially like when you know, understanding what are the problems that the patient encounters in their daily life, how can we, you know, clarify their misconceptions about certain health, yeah.

Interviewer:

Okay, so put what you've learned in the curriculum into practice, right?

Student:

Yeah

Interviewer:

Okay, so in general, what elements of teaching and learning in pharmacy curriculum like the design of the modules, the projects, the teaching mode, assessment, teaching staff or learning environments, etc. that you think have an influence on the promotion of health advocacy among pharmacists?

Student:

Wait, so which is again? What are the pharmacy module aspects?

Interviewer:

Yeah, what elements of teaching and learning in the pharmacy curriculum? So like the design of the modules, the projects in the modules or the teaching modes, online or physical, study in the lab or like assessments or the teaching staff or the learning environments, which one have an influence on the promotion of health advocacy among pharmacists?

Student:

Oh okay, this one right. I think I will mostly say, uh, is the things that is conducted outside of the pharmacy modules, so things that is like the program that I just mentioned earlier, the compulsory volunteering the mandatory program, the interdisciplinary program, like that, those are the most important

aspects of the modules. Because other than the right, the rest of the module is just.... They just teach us like it's just classroom, traditional classroom setting and they didn’t really touch upon much about health advocacy, so it's all like just like fundamental knowledge, like anatomy or diseases and so on, there's nothing about health advocacy.

Yeah, I don't think there's much, because to me, I think health advocacy is you have to really learn it by interacting with patients. It's very difficult to learn in a classroom setting.

Interviewer:

Okay, so how should these elements of teaching and learning be used to deepen the understanding of health advocacy among pharmacists?

Student:

Uh, how much ah?

Interviewer:

No. How should these be used to deepen the understanding of health advocacy among pharmacists?

Student:

Oh okay, I was thinking about like the other questions, so I lost track. Yeah, so how should they be deepened ah? If I'm not wrong, I mean this question is very hard for me to answer 'cause I'm a student, not a module coordinator.

Interviewer:

So what you think should be changed in the elements of teaching and learning to like improve the understanding of health advocacy among pharmacists?

Student:

Then then maybe I have a simulated classroom so like they have simulated patients or just to … or better, they can actually dedicate one lesson, or not dedicate one lesson. They can give us like a rough introduction of health advocacy because, like so far, what I learn right, my modules didn't really explicitly mention health advocacy. I don't think they just ... maybe very subtle, the way they teach us.

So they don't really... you know, it's kind of there, but it's also kind of not there, you know.

Interviewer:

Oh, okay.

Student:

Maybe certain basic health advocacy knowledge could be given. So for example, like, uh, okay, they do give us non-pharmacological intervention. That's what I quote, the proper term, that just means a lifestyle changes, which I believe is a part of health advocacy.

But that part is not really, they didn't really explicitly mention that as under health advocacy. Yeah, they just put it as together with like the medicine and the diseases. I don't know whether you can really call as health advocacy. Maybe if they explicitly highlight it first and then they tell us what is the importance of health advocacy as well as some basic things that you can say to them you know, like cover it like a actual lesson itself so that we have better awareness of health advocacy.

Interviewer:

Okay, I see.

So, next question is what elements of the core curriculum offered by the Department of Pharmacy, for example the co-curricular or enrichment programs or you think have an influence on the promotion of health advocacy among pharmacists? I think this question you have answered, right?

Student:

I think so. It’s the volunteering in the LP.

Interviewer:

Yeah, okay, so how might this element of the co- curriculum be used to deepen the understanding of health advocacy among pharmacists? So what do you think that these co-curricular or enrichment programs can be improved?

Student:

Actually I don’t really know, I think it's okay. Also it's quite hard to improve because to me, different students experience different things. They have different teammates, different patients, so they obviously have different, you know, different levels of health advocacy.

Yeah, that’s what I would say, so it's not really one size feet.

So let's say you suggest an improvement, right? It may benefit some students, but it may not benefit others.

Interviewer:

Oh, I see.

Student:

Yeah, 'cause you have advocacy is very individualized.

Interviewer:

So to you so far, these programs are okay, good enough?

Student:

Yeah, yeah, it indirectly helps me to expose what is what health advocacy is like.

Interviewer:

As the new pharmacy curriculum is very much based on basic, clinical and system sciences integration, was this integration apparent to you and does it contribute to your understanding of health advocacy?

Student:

Integrated right? I would say yes, it's quite integrated, especially in the second semester. But does it relate to health advocacy? Yeah, I'm going to say no, not a lot.

Interviewer:

How?

Student:

Okay, so there’s clinical sciences right? Yes, there's clinical sciences. There is a physical sciences. It is, uh, what's the third one? I've forgot already.

Interviewer:

Basic clinical and system sciences integration.

Student:

Uh, so basic clinical and system right? So basic is like.... I believe it's just chemistry and so and yeah, so system is like human body, if I'm not wrong, anatomy and all these. And clinical will be a problem information, like pharmacists, like what the pharmacists do, essentially, so they combined all these 3 together.

Student:

So the only way you can find health advocacy for this syllabus, however, health advocacy, to me, strictly right is under clinical.

I don't think I can find it in basic or system. Because there's no point for it being in them ma, right? Well, I mean, the department didn't see any point in, you know, putting them there, it just it just put under like somewhere under clinical and it is also not very directly like went through that so I didn’t even know there's this thing in health advocacy until after I met a lot of patients and talked to them that this story slowly comes to my mind.

Other than that, I didn't pay attention in class, but I don't think it's a significant thing in the modules. I don't think so.

Student:

Because we are not tested it for our exams. So other than the non-pharmacology intervention, that one we are tested, but it's a very small aspect of the whole thing.

Generally, we are tested on our communication skills, our other stuff that health advocacy is not a major criteria. Yes, so I would say that it is not actually very heavily focused on health advocacy, I think due to like certain reasons that the department has.

Interviewer:

So how do you think that the department can improve on this integration?

Student:

I think the first question they must ask is: Is there a point in this integration?

Oh yeah, or like, is it feasible not to do this integration? Because how are you gonna do health advocacy in basic science or system sciences.

To me, I think it's quite difficult. But to me the best is really just going out there and practice with patients, that's how health advocacy comes naturally. Instead of like me having to learn it in class and memorize, it's not very organic.

Interviewer:

Okay. So what kinds of modules or programs or activities related to the promotion of health advocacy would you expect to experience in your second year?

Student:

Hey sorry, what's the question again?

Interviewer:

What kinds of modules, programs, or activities related to the promotion of health advocacy would you expect to experience in your second year?

Student:

My second year, definitely more non-pharmacological intervention, lifestyle changes I think. I mean, I'm not saying it's bad ah, because I- I don't mind, but I think health advocacy is very nuance. It is not something you can just teach students, so probably I would expect maybe more patient visits if possible.

Yeah, 'cause given the situation now, I think it's quite helpful, so it's all online.

As far as maybe making more explicit, like cover that, what is the basic principles of health advocacy, so like because I think to us, the students, we don't really bother if anything that is not tested. I think that's a very well-known fact like: it's not tested, I'm not gonna go and look at it, not gonna bother. I'm not gonna go and understand it.

So I think so. Maybe make it examinable. Yeah, maybe I don't …. see first. I'm not very sure, but I know that if it's not assessed right, then students will not bother with it.

Interviewer:

Yeah, sure, so I just asked about what would you expect. So now, is what kinds of modules, programs or activities related to the promotion of health advocacy would you personally like to see or experience yourself? And can you give some examples?

Student:

Oh, health advocacy... maybe a short lecture about what does it mean to be a health advocate.

Okay, I think I did that in 1150. I think I did that in my presentation. But well, I think it's very surface level to me. I didn’t learn that much about health advocate.

Umm yeah, I mean okay, Dr Koh and Dr Han went through a bit but maybe they can have a bit more guidance or elaboration like in the next module, so 2150.

Interviewer:

Oh, so you expect health advocacy to appear in higher level modules?

Student:

Yeah, high level modules.

Interviewer:

Oh okay, I see.

Interviewer:

So that's about it. Thank you so much for answering all the questions, yeah.
