# Supplementary material for: Evaluating the internalisation of the intrinsic role of health advocacy of student pharmacists in a new integrated Bachelor of Pharmacy curriculum: a mixed-methods study
Source: BMC Med Educ. 2023 Nov 27;23:900. doi: 10.1186/s12909-023-04877-y (PMC10680209; doi:10.1186/s12909-023-04877-y)
Supplement: Supplementary file 2 — Additional file 2. [file 12909_2023_4877_MOESM2_ESM.zip › Raw Data/Post Year 1 Interview Transcripts/Post Year 1_Interviewee 2_Transcript.docx]

# Transcript of Post-Year 1 Interview with Interviewee 2

Interviewer:

Um, so the first one is, uh, what role do you think that pharmacists have to play as health advocates in Singapore society and can you give me some examples?

Student:

Okay, so I think that we play a quite... How do you say it... underappreciated role as health advocates.

Yeah, because as health care professionals, I think pharmacists naturally do play a role in actually promoting to the public about upkeeping of health. So I think a specific example would be like, how they involved in smoking cessation programs like pharmacists led smoking cessation programs that actually are part of the health advocacy program. So I think that is one example that shouldn't be neglected.

But yeah, I can’t think of other examples right now, but I'm sure there are many ways that we pharmacists are actually involved and primarily, our role is like medication experts, we play an important role in making sure, promoting health through the use of medications, yeah.

So that's also another important role that we play as health advocates, and I think that's why some more attention should be placed. In the future, I hope more responsibility can be given to pharmacists to really exert their role as health advocates in the society.

Interviewer:

Okay, thank you so much.

How might you see yourself as an advocate of good health in future as a pharmaceutical or healthcare professional?

Student:

Okay. Okay, so I think that's a good question. Let me think about this.

I think that firstly, I hope that the ministry gives us more freedom and responsibility to advocate for health. I think that will increase our scope of what we can do as pharmacists, 'cause right now, what I seem to understand from the role of pharmacists is that in the clinical setting, there're still a lot of restrictions on what we can do, so that will also limit our ability to advocate for health in the larger society. So I hope moving forward that more emphasis can be placed, more can be given to pharmacists to advocate for. I think that moving forward, if that’s the case, then we will be definitely able to do more, also because of like the whole like automation and IT, we can really leverage on that and do things more efficiently.

Yeah, so uh, as of now I'm not really sure of completely what we can do because I was not too sure, I'm only year 1 so I'm still learning but this is, I think the direction that I think that we should also be looking to.

Interviewer:

Yeah, okay, yeah, very interesting. So what three characteristics best describe an effective health advocate to you?

Student:

Effective health advocate? Let me think about it. These are good questions by the way. I'm not sure I can give a good answer.

I think firstly, an effective health advocate should be reliable, so that someone in the public can trust, and can do the job well, advocating for health.

I'm not sure whether passionate would be the right word, but like really, I guess, motivated to advocate for health. Like you know, someone who really lives by example, who is not reluctant to do it, but really wants to and desires for actually health advocacy, drugs across the community.

The third one, I think this sort of overlaps with reliable. I was saying knowledgeable because I need to know that you need to be well aware of how to best advocate for health and what is actually needed, yeah. That's like the first. You need to have the knowledge before it can be reliable and before you can be passionate.

So yeah, I think I think that's what I can think of now, yeah, but.

Interviewer:

Okay, that's a good answer.

Okay, so do you feel that you have a basic grasp of what health advocacy entails? Are you ready to move on to the next phase, and why or why not?

Student:

I feel like I don't know that much. I hope I’m not disappointing the professors.

I still have a lot to learn, but from what we've covered in year one, I think they're trying to impress on us that it's not just about providing health care but also managing health, just to prevent people from even in being the state to need health care services. So it's like keeping people healthier, basically.

That’s the main gist of how I understand health advocacy, and how they have tried to deliver it to us. I think there are a lot of details that we are still going to learn and to explore in the years to come. Yeah, but for now I think that's what I understand.

Um, what's the 2nd part of the question?

Interviewer:

Are you ready to move on to the next phase?

Student:

What's the next phase?

Interviewer:

Based on your understanding.

Student:

Uh, us being the health advocates, not generally, but health advocacy?

Uh, I guess I'm curious about next stage. I feel like I need to immerse myself in health advocacy more, you know, because now we're just learning about it, and we haven't really got the chance to experience it.

I think so. I think more practice and more of it being put in like, maybe we get more experience than before, definitely more ready as health advocates.

Interviewer:

So you are expecting for more practice in health advocacy?

Student:

Uh, yeah, I’m hoping.

Interviewer:

Has your understanding of health advocacy changed after your first year as pharmacy undergrad?

Student:

Oh okay. In the beginning I didn't know much, so it's not a very great benchmark to compare with, but yeah, I guess.

I mean of course we all know like the basic meaning of health advocacy if you break it down into its literal meaning, but I think being in the program makes it more unique and also because it's like we are going to be part of this health advocacy thing, you know. There's a difference when we are learning with purpose, learning health advocacy with a purpose because we are going to be the future health advocates. So I think that that made us have now a slightly better understanding and more perspective to what pharmacists can do in the future as health advocates.

Interviewer:

So before entering uni, you haven't known about this… you didn't know about health advocacy, but now you kind of get some basic understanding right?

Student:

Uh, to add on to that, I can really see more of the role that pharmacist can play in health advocacy. That was the main path.

Interviewer:

Okay, thank you. To what factors would you attribute this change in your understanding of health advocacy over the academic year?

Student:

Uh, I think to put the change to just something alone, it is difficult. I think it's mainly joining through the whole year, going through the curriculum, especially 1150, I guess, for lots of perspective, what we can do as specifically as health care professionals, how we can advocate for health, and also I also appreciate that like Dr Koh and Dr Han Zhe actually really ... I don't know whether passionate is the right word, but I guess they really enjoy talking about it and you know they really lead by example.

I guess I can sense that oh, you know, they're really advocating for what they're talking about, you know, so I guess that kind of interaction is inspiring and also made me understand health advocacy a bit more.

Uh, I think if I have to pinpoint to like what caused the change would be probably that, yeah.

Interviewer:

Um, did you participate in any cocurricular activities or enrichment programs that is related to health advocacy?

Student:

I'm not sure whether this is counted as related to health advocacy, but there’s this virtual befriending thing that I signed up for, so it's actually we get to interact with some elderly and then we get to advocate to them about health, yeah, so we talk to them and then learn more about their conditions.

It was like a three week, three times, basically. So first time we just get to interact with the elderly, and the second time we actually prepared a presentation to come and talk to the elderly about like how to improve his health. And yeah, I guess that can be counted. Even though there’s only students and of course much limited in the capacity of what we can really advocate for. So just an example, because the elderly was actually not taking his medications, yeah, so I don't know how whether it's effective, but we did convince him to take his medications for cholesterol. And because he didn't want to get medications, other than medication, he was fine, but just that one, so we advocated for that. And also, I'll talk to him about good habits like exercising and stuff like that. So in our own capacity, that was what we could do.

And I guess that was kind of it, yeah. In the future we will get to do like more than this and we really get to impact lives, yeah, so getting to experience it is really fulfilling that, yeah.

Interviewer:

Okay, interesting experience, right?

Uh, okay, so did that activity change your understanding of health advocacy, or did it like to some extent, affect what you feel about health advocacy like they make you like to learn about health advocacy more?

Student:

Yes, I would say yes. It triggered that, like spark, I don't know. Yeah, it just made me feel like I want to do this more.

Yeah, because like seeing how our words, our care for that particular elderly could … actually it’s just ask him do things, medications, for example, to incorporate these practical exercises in his daily routines. It doesn't seem like much, but like we know that it's going along with, and we can see that in how the elderly response us, and then we know that he understands that we care for him and we want the best for him. Like if we are able to do that for more people right in the future, I think that's quite inspiring for me, that's why I think it really excites me to be learning more about health advocacy and actually doing more of these, getting more experience.

So I guess that's my answer.

Interviewer:

Yeah okay, thank you and, uh, okay, so going back to the question of the change in your understanding of health advocacy over the past year. And so what you learned from the modules, some other activities, uh, was it aligned with your initial understanding of health advocacy before you enter uni?

Student:

Yes, yeah. I don't really know how to answer this question. Yeah, is it aligned? I guess yes. I don't see how it cannot be aligned.

I don't see how it can be misaligned. I mean, unless I totally did not have any impression of health advocacy or had wrong impression of health advocacy, maybe it might have been misaligned.

So that's all I can say, yeah.

Interviewer:

Okay, so in general, what elements of teaching and learning in pharmacy curriculum like the design of the modules, the projects that you did, or the teaching modes assessments, teaching staff or the learning environments would you think have an influence on the promotion of health advocacy among pharmacists?

Student:

I guess from the new curriculum has a lot of changes, yeah, but specific to health advocacy, I think because we get a lot more clinical experience, which means that we meet with patients and we learn about clinical skills, the ASCII. It just impresses on us the importance of the role we have as pharmacists, oh, in the health care setting, how important the role that we play. Yeah, as compared to the previous curriculum.

That's why I think because of the different assessment, like ASCII and the way that they teach is integrated. So there's a lot of skills, not just knowledge, but skills. And then we have 1150, which is, like the professionalism, the attitude and stuff like that. It's just reminds me of a very holistic learning and because of that, I think it empowers us to become like, not only be excited about a future, but also can see how important a role that we can play.

Yeah, because I think sometimes we can regret. Like some of us may feel that pharmacists may not play such an important role in the health care setting, especially in health advocacy. So like with this new curriculum rolled out, Year 1, you are already introduced like how important and what kind of role you play, and then we can build on that in the future, yeah, that's what I feel.

Interviewer:

Okay, so how might you think these elements can be used to improve the understanding of health advocacy among pharmacy undergrads?

Student:

I guess they can make it more explicit, I don't know. I don't really have much to say about that.

I don't know what this might be quite subjective, but I think it would be good that for example, for me I think that it would be good that I know that I'm what I'm learning, like I'm aware that I'm learning about health advocacy, not just like put in the experience and I'm expected to take away what health advocacy is, how it would advocate for health. But I'd rather it be explicit, like they told me. Yeah, I'm not sure whether it's the same for everybody, but because when you explicitly told me I can be more purposeful, I will be thinking about “Oh! This is actually health advocacy and like this is how we do it and that's how we can be effective.” I guess that's the only thing I would say can be improved.

Yeah, because it’s the new curriculum, everything is integrated, so maybe that may not have been so as explicit in the health advocacy thing, yeah, but I mean I do see the traces, of course, yeah.

I do see it being implemented in the curriculum, but just that it would be great if we can tell you that “Oh! You know we're doing this because we want you to learn.” I think that that would be great for me.

Yeah, I'm not sure about other people.

Interviewer:

Yeah, I think it's a quite general feel of pharmacy undergrads about this, like the health advocacy knowledge in your curriculum right now is still quite minimal and is not tested.

Student:

I mean it doesn't have to be tested. I mean, it's hard to test about health advocacy. It’s like a skill you know. It's a holistic skill. You can't just be tested, but I think if they make it more explicit and have more frameworks that guide us, that kind of thing. Yeah, I think that would be quite helpful.

Interviewer:

As the new pharmacy curriculum is very much based on basic, clinical and system sciences integration, was this integration apparent to you and does it contribute to your understanding of health advocacy?

Student:

Uh, it was very apparent, yeah.

I chose the course because it was. I guess it's same thing, it impresses the importance more than the previous curriculum, about health advocacy.

Interviewer:

Yeah, okay, so the new one is better in terms of integration into health advocacy, is it?

Student:

Yeah, I think so. I mean for me, I can't speak for everybody. I don't know. Maybe somebody who might have completely different views. So I can only say for myself.

Interviewer:

Yeah, okay, so how can the department improve on this integration, you might think?

Student:

Um, is this specific to have advocacy, or like the whole integration?

Interviewer:

Yeah, yeah.

Student:

I think other than making it explicit right. Yeah, I think I think that would be the main issue for me 'cause I don't really have much of an issue with the program and I like that it's promoting health advocacy.

But sometimes it can be, maybe because of the integration, it can be a bit messy. It can bound to be a bit messy, like suddenly we're learning from here, then there, then like, maybe a jumble. And because of that, maybe health advocacy isn't that clear to us.

That I would say that integrating is definitely not bad thing. This is specific to have everything in order for us, the students to be very aware of it and be able to improve our advocacy skills in the future.

It's important that it's made very clear to us. From what I know, it's just been like we put into some parts here, some parts there. Yeah, so sometimes it can be a bit blurry. Yeah, so if it was more clear, it would have been better.

Interviewer:

So far, it very much depends on how those students understand and how they…

Student:

Yeah yeah, correct, correct. I think what you mentioned is so true. It's just our own interpretation. Yeah, correct. So it's like the professors may not actually know how we interpret.

Yeah, yeah, I’m not saying that they have to test about health advocacy but because it may not have been made so clear, I'm not sure if every pharmacy student is aware that we are talking about health advocacy.

Yeah, they may have different perspectives on it, may not be exactly what the professors want us to know, yeah.

Interviewer:

Okay. So what kinds of modules or programs or activities related to the promotion of health advocacy would you expect to experience in your second year?

Student:

I don't know if we had the opportunity to do this in… okay, I think in year 1, we didn't have an opportunity to do this, but like during our professionalism work which is 1150, which is next year, will be 2150 right? I hope that at least that you get like good number of chances to really experience, like case studies about health advocacy, like the ones that we've been doing year 1 was all about ethics and professionalism. Yeah, I won’t say that they're completely irrelevant to health advocacy but it's mainly... everything is like the ethics and values, yeah not really the health advocacy part. Then again, it may not be the aim of the professionalism module, maybe saying that... maybe it can be added somewhere, I don't know ... maybe the skills module, like a specific segment about advocating for health can be added.

I think if there's a specific segment about health advocacy, I know they may not be able to teach that since like it's a skill that needs to be built on.

But we know that “Oh! There is a skill called health advocacy and like it's important to us.” Yeah, because it's made to be a segment. I know it sounds kind of bad. Well, I think that'll be… that'll make things a lot clearer and the emphasis will be there.

Interviewer:

So this question is about what you personally like to see or experience. So the difference is expect and personally like to see or experience.

Student:

Oh okay, okay. Oh, I see. So personally like I guess. Uh, I would really hope to actually practice health advocacy. Yeah, for example, the virtual befriending, more of these opportunities to us, because it's something that I enjoy, I guess.

And I think with more practice, we will slowly build on the skills. Yeah, it's something that just lecturing won't help. Yeah, something that you experience and then you build on that. So yeah, that's what I would personally want.

Okay, not much different from what I expected.

Interviewer::

More practical activities, right?

Student:

Yeah.
