# Supplementary material for: Evaluating the internalisation of the intrinsic role of health advocacy of student pharmacists in a new integrated Bachelor of Pharmacy curriculum: a mixed-methods study
Source: BMC Med Educ. 2023 Nov 27;23:900. doi: 10.1186/s12909-023-04877-y (PMC10680209; doi:10.1186/s12909-023-04877-y)
Supplement: Supplementary file 2 — Additional file 2. [file 12909_2023_4877_MOESM2_ESM.zip › Raw Data/Post Year 1 Interview Transcripts/Post Year 1_Interviewee 3_Transcript.docx]

# Transcript of Post-Year 1 Interview with Interviewee 3

Interviewer:

Okay, so the first question is what role do you think pharmacists have to play as health advocates in Singapore society? Can you give me some examples on at the individual or population level?

Can you give me some examples?

Student:

Okay, yeah. Actually, when I first heard the question right for health advocates, what I was thinking was like how you promote health as pharmacists basically. So yeah, when you talk and dispense the medication to the patient you can maybe like recommend how the patient can improve the health, promote health and prevention stuff like that.

Then actually, I wasn't very sure on what a health advocate is, so into Google, but I'm not sure like whether I should be Googling for this interview, but I just went to Google and what I saw was like there's this definition “health advocates give patients and their families direct customized assistance in navigating the healthcare system”. So I'm wondering like whether the health care advocate helps the patient to access health care based on this definition.

In that case, I guess as pharmacists: Let's say the patient comes up to us with a dry eye, we can first help to recommend the patient with some eye drops, but then if it doesn't improve in a few weeks, then we recommend the patient to go and see a doctor, so I guess it’s helping the patient navigate the health care system and also the definition also includes educating patients so that they can make well informed healthcare decisions.

So I guess this can also be done by pharmacist by like presenting the numerous drug therapy options, and like seeing whether the patient wants the one that is like cost effective, like lowest costs or like one that has less side effects but higher costs and see what the patient wants, yeah?

Interviewer:

Okay, thank you so much.

Okay, so the second question is: How might you see yourself as an advocate of good health in future as a pharmaceutical or health care professional?

Interviewer:

So, how might you see yourself in those roles? And as an advocate of good health and how you promote health advocacy if you are in the roles that you mentioned?

Student:

Okay, for me right, I would really like to work in patient care in the future, maybe in like the polyclinic or something.

So for me, I think I'm gonna go like more broad-based, in a sense that I'm thinking of the way that health advocacy work. So my definition of health advocacy would be to promote health.

So promoting health, right? I'm thinking like this prevention and maintaining health and not letting it deteriorate further.

So for pharmacists, I think our role is bigger in the maintaining health and not letting it deteriorate further, 'cause we don't really reach out for people with good health and for prevention, because normally when they come to us right, they have a prescription, they have some condition and we're already treating them. So like their health is not at the best. So yeah, I think my role next time will be more towards the maintaining health, not so much on the prevention.

And I think the role is not very big. In the sense that I think, like perhaps advertisements online have a larger reach and impact than what pharmacists are telling the patient. But I think we still have an important role in educating the patient.

Interviewer:

Okay, thanks for your answer and the next question is what three characteristics best describe an effective health advocate to you?

Student:

Can I just say, like knowledgeable or something? It must have done knowledge to be able to help the patients.

I was gonna say the word convincing, but then [the other interviewee] said in his last sentence. But okay, convincing 'cause like yeah, you have to be able to tell the patient why they need to do this and so that the patient can listen to you. So in a way, kind of need to be like a people person so that you can get the patient to listen to you.

And so I said like knowledgeable, you have to know and you have to be convincing also.

I guess maybe my last word can be like… okay, I guess like I can say the word patience. Because sometimes it's not very easy to convince someone. But like yeah, patience in that you can explain the reasoning why? So they can communicate with the patient even though it might be hard.

I'm so sorry that was not good.

Interviewer:

I think it's a good characteristic of health advocate, yeah?

Okay, so do you feel that you have a basic grasp of what health advocacy entails? Are you ready to move on to the next phase and why or why not?

Student:

What do you mean by the next phase?

Interviewer:

Based on your understanding.

Student:

So for me, I will also say that I don't really have a basic grasp of health advocacy, given that I also needed to Google the term health advocacy just now.

Yes, and for the new phase, but maybe I was thinking more of like the transitioning, more into primary care of Singapore. So like maybe pharmacists will have a bigger role in health advocacy and in primary care.

I would say that I would welcome this change for pharmacists to have a larger role in the health care team, to the public other than just dispensing medication, and I would welcome this new phase. I am ready for it.

Interviewer:

Has your understanding of health advocacy changed after your first year as a pharmacy undergrad? If yes, to what extent has it changed?

Student:

Sure, I would say that there hasn't been much change in my understanding of the word “health advocacy”.

Perhaps before I would just think like health advocate, like someone who advocates for health, someone who promotes for health and my understanding before I googled it was pretty much the same thing. But I think after Googling it, I think I see a connection with what I learned in year one in the sense that it was kind of highlighted to us that pharmacists play a role in promoting the health of people, especially from maintaining health.

Interviewer:

Yeah, okay. To what factors? Let's say the factors are the curriculum, the teaching staff, CCAs, or enrichment programs. So to what factors would you attribute this change in your understanding of health advocacy to?

Student:

Can you repeat the options again?

I would say like the curriculum. The curriculum definitely, and the teacher that come along with the curriculum.

And then of course, the curriculum is quite basic, they would just go and ask what is health advocacy, explained to us the importance and whatnot. I guess the teachers is that their attitude in class when we ask them questions, they are very passionate about their work and they really try to explain to us like why we should do this and the purpose of this and this, which really shows us our roles in promoting health. Because they are trying to do it as well.

Interviewer:

Okay yeah, the next question is, in general, what elements of teaching and learning in pharmacy curriculum like the design of the modules the projects are teaching, modes, assessment, teaching staff or learning environments or you think have an influence on the promotion of health advocacy among pharmacy undergrads, yeah?

Student:

Yeah, Oh my gosh, that's a lot.

Interviewer:

You can choose to talk about one or two or whichever you like to talk about, yeah.

Student:

Okay, so actually maybe I will also talk about module, but PR1151, it’s where we get to experience patient care. I don't know how to explain it, we learn how to interact with patients and talk to patients. So this kind of ties in with a mode of teaching that I think it's very good for health advocacy, which is like to actually see how it is done by professionals, our professors who are pharmacists as well.

Not only do we learn how to interact with patients in that sense and I thought like what to tell the patient, we also see from our teachers in real life because...

OK half online half in real life, but we got to see how they actually faced towards the nuances and how they interact with patient basically and show the characteristics that we said just now of being convincing and knowledgeable and applied all that in order to promote health advocacy. So I think that that is very good for our understanding of health advocacy.

And I think like if I can summarize like both what me and [the other interviewee] were talking about modules, I think like the way that they designed the curriculum was very good because all these modules kind of like integrated together, which help us see and understand like how it all linked to us, being able to promote, maintain good health.

Interviewer:

How might these elements be used to deepen the understanding of health advocacy among pharmacists?

Student:

Oh wait, let me see. Let me type health advocacy in my notes.

Student:

Okay, I think for me, what I would say right is maybe really like get into that of what is health advocacy and how we choose to we show it, because when I search in my notes, surprisingly, it's not under 1150 unless I said from.... from what I see, it is like … in 1153, it says like not really specific under health advocacy, but it's under like infectious agents, so it says like “practice and advocate infection prevention and control...be an advocate for immunizations” and stuff like that. So I think that having a clear definition of what is health advocacy and it being integrated into our curriculum in different modules like 1153 when it appears then they can remind us again that this is health advocacy. So maybe at the beginning of the year, has a more in-depth introduction to health advocacy so we can understand it and then have these reminders along the way.

Interviewer:

Oh, I see. So you want it to be mentioned more specifically and a bit more explicit.

So the next question is about the elements of curriculum offered by the Department of Pharmacy. What elements of co-curriculum would you think have an influence on the promotion of health advocacy among pharmacists?

Student:

Okay so I'm not sure whether this is counted as co-curricular activities, but I would say like the webinars that the department, The Pharmaceutical Society organises, they can help in promoting health advocacy.

I think maybe for specifically there's this committee, the Pharmacy Profession Awareness Committee, they like help link the importance of pharmacists to health advocacy and help us see in what way pharmacists can play a role in health advocacy and maybe some events that they do will help in that, yeah.

Interviewer:

So, as the new pharmacy curriculum is very much based on basic, clinical and system sciences integration, was this integration apparent to you and does it contribute to your understanding of health advocacy?

Interviewer:

And how does it contribute to your understanding of health advocacy?

Student:

Okay so for me, I think I'll touch on the integration in like more of a patient care manner. So the things that we learned in the science modules we got to apply it through our patient care module, so when we actually talked to fake patient and we use the information that we learned from our other modules, so it was linked that manner and I think it was very good in my understanding of health advocacy because it not only deepens my knowledge of what we learn in the science modules. Because we get to actually practice it with a patient, but we also get to practice how to phrase it in a manner and actually see how we can use the knowledge to address the patients concerns and help the patient in maintaining their health.

Interviewer:

So how can the department improve on this integration?

Student:

I think for me, I would say like maybe the teachers are not very used to the integrated style yet, because they haven't been in the program for very long, so sometimes they just like skim pass things and say like you have learned this before, but they don't really tell us like where we have learned this.

So like, let's say for example, I think in one of our modules, the professor, always says like you will learn this in Year 3, so we don't really look at it at all, but I'm wondering like whether we should at least take a look at it because I'm scared that in Year 3, the professor will be like, “I think your professor mentioned this in Year 1”, then I'm scared like we wouldn't have the correct link.

And I think if one professor taught the Pharmacy Foundation module, and another professor is teaching another module, that touches upon the basic stuff in the Pharmacy Foundation module. I think that maybe the professor can look at what was taught in the Pharmacy Foundation module and have a better understanding of what we understand from that module.

Interviewer:

Okay yeah, I see.

Interviewer:

So the last two questions, what kind of modules, programs or activities related to health advocacy would you expect in your second year?

Student:

I think I would agree with [the other interviewee] in the sense that I don't really know what is health advocacy, so maybe this sharing, the profs may want to like … at the start of Year 2, maybe bring up health advocacy and the importance of it so that we have a better understanding.

But I think the rest should be quite okay, like having the modules with some stuff sprinkled into them about health advocacy is good, then bringing up to us again that it is about health advocacy.

Interviewer:

Okay, so in a way it should be more explicit, right?

Student:

Yes and emphasized.

Interviewer:

Um, so more knowledge about health advocacy, right? Okay, so uh, that's about what you would expect. I'm not sure whether it makes sense for this question, but I want to ask what kind of the modules, programs, or activities that you would personally, like to experience.

Student:

Can I throw something like out of the blue?

I think for us, during our like tutorial sessions, sometimes we have these kits, and the professors are testing to see whether they are useful for learning. Personally, I think that is quite useful for me because instead of like reading what to do, we actually get to see what is happening in a situation, so I think more of these sort of kits can be useful in learning health advocacy because like I said, we don't really have a very good understanding of what is health advocacy, so if having this kits, the professors can show to us like “Oh, in this situation, you can actually display health advocacy. I think that that's quite useful”.

Interviewer:

So, maybe more practical activities and some case studies, some scenarios for you to practice?

Student:

Yes, yes.
