# Supplementary material for: Evaluating the internalisation of the intrinsic role of health advocacy of student pharmacists in a new integrated Bachelor of Pharmacy curriculum: a mixed-methods study
Source: BMC Med Educ. 2023 Nov 27;23:900. doi: 10.1186/s12909-023-04877-y (PMC10680209; doi:10.1186/s12909-023-04877-y)
Supplement: Supplementary file 2 — Additional file 2. [file 12909_2023_4877_MOESM2_ESM.zip › Raw Data/Post Year 1 Interview Transcripts/Post Year 1_Interviewee 4_Transcript.docx]

# Transcript of Post-Year 1 Interview with Interviewee 4

Interviewer:

Okay, so the first question is what role do you think pharmacists have to play as health advocates in Singapore society? Can you give me some examples on at the individual or population level?

Student:

So how do they act as health advocates.... so pharmacists are known as the chemists in healthcare, so they are in charge of the different drugs, from what I understand and we can advise the patient. Let's say if they come to a pharmacist and they say they have these symptoms here and there, what we can do if it's not as severe, we can give them certain diagnosis and we can give them certain medication if it's not too severe, so our job is especially this medication and what we'll do is we'll … And also we are part of the primary care, so anything that hospital can't do, so we can handle things that, uh, let's say they don't have to go to hospital to actually do. So we provide primary care. We will reduce the number of cases, if not hospital will be overcrowded. I think that will be my answers as health advocate. So individual-wise, it's primary care. Sorry, what was the second part?

Interviewer:

Can you give me some examples? Actually you just did it.

Student:

Yeah, I think I answered some of them.

Interviewer:

Okay, thank you so much.

Okay, so the second question is: How might you see yourself as an advocate of good health in future as a pharmaceutical or health care professional?

Student:

Well, I would. I would say that after completing a bachelor in Pharmacy, I think my goal is to go in to healthcare sector first, to try out certain specializations in pharmacies. So one of my goals would be to try out on the oncology, because I think it's very interesting.

I don't really know much about it because what we learned so far, we learnt only the very tip of the iceberg for oncology, about certain things like carcinoma and melanoma, and I'm still very interested to explore this sector here.

Student:

Um, following this healthcare sector, maybe I would like to go to sales because I feel that I'm a rather people-oriented person so sales is something that is of great interest to me.

I would like to go into the pharmaceutical industry and I can see myself on maybe going doing sales, going to hospitals, and then I'm trying to sell the products of my company.

Yes, so these are the two sectors are very interested in. I can see myself going into.

Interviewer:

So, how might you see yourself in those roles? And as an advocate of good health and how you promote health advocacy if you are in the roles that you mentioned?

Student:

How do I promote health advocacy?

Uh, I would say that by encouraging non-pharmaceutical lifestyle choices. For example, what we learn about dry eye to take more omega-3 products, to take more supplements, and especially for ARMD, this disorder, you can take things like supplements, some vitamins, vitamin C, vitamin E, Lutine and all. Like that and also exercise more, take more vitamin E here and there. So I think as pharmacists, we can encourage this kind of lifestyle choices, maybe especially to quit smoking, that’s one very, very big factor indeed.

So I think these are some things that I would advocate health in a way.

Interviewer:

Okay, thanks for your answer and the next question is: What three characteristics best describe an effective health advocate to you?

Student:

Maybe this is 1150.

I think integrity will be one. Can I just list out one by one?

Interviewer:

Yeah yeah, sure sure.

Student:

Okay, I would say very passionate in helping people. If you have a passion for something, you then go out of your way to help them. So, especially if it comes to promoting health. So if you are very passionate about it, you will tend to help more of your patients, yeah.

Interviewer:

Do you have any other characteristics?

Student:

I think as health advocate, you need to be inspiring. You need to inspire patients. I mean you need to motivate your patient, maybe motivating is the word I'm looking for.

You need to find causes, find reasons for why your patient should be doing this, why are they carrying out these preventive measures here and there, or maybe these maintenance measures? So I think it's all very important.

You need to be somebody who's very motivating, somebody who is able to persuade, to convince, especially when it comes to medication adherence, that’s very important. Those are some of the errors that pharmacists face, not really errors but in a way - problems that pharmacists face. So being motivational, being convincing, persuasive is a very, very key factor as well.

Interviewer:

I think it's a good characteristic of health advocate, yeah? Okay, so do you feel that you have a basic grasp of what health advocacy entails? Are you ready to move on to the next phase and why or why not?

Student:

Can you repeat the first part again?

Interviewer:

Uh, do you feel that you have a basic grasp of what health advocacy entails?

Student:

Okay, this is a challenging question, but I will give it a try.

Student:

So I would say that. Nobody is perfect, so I would say that.

Having a grasp of what health advocacy entails, I wouldn't say that I reached there yet. Maybe on the way because I'm still in year one and I am always learning. I feel that it's a life-long journey.

I believe that this Bachelor of Pharmacy program, they will give me many more opportunities to practice patient care and to show care and empathy to people, especially in assessments-wise. After every assessment, I feel like I learned something new, something that I didn't know before the assessment. So definitely on the part whether I have a grasp of it, I don't think so, not a total whole of it yet. And moving on to the new phase, for my understanding is basically...

There's two things that are coming right now, which is either year two or graduation. So I only had one year in the pharmacy and I don't think that I'm ready to graduate and.

Even though I'm very proud what I've learned and all, and I can share the knowledge, the skills to my parents, to my loved ones, I don't think I'm really there yet.

Whether they will move on to year 2, I believe that the hard work in Year 1 should more or less prepare me for Year 2, what the professors have given all the materials and all.

I think they're very, very good. I like how the new curriculum is going as well. I can see the integration right now.

I'm moving on to the new phase, so I would just like to believe new phase as in year two. I like to think so.

Interviewer:

Has your understanding of health advocacy change after your first year as a pharmacy undergrad? If yes, to what extent has it changed?

Student:

Can you rephrase the question? I don’t really understand.

Interviewer:

Okay, so how does your understanding of health advocacy change?

What's the difference of your understanding of health advocacy when before you enter uni and now?

Student:

Uh, likewise. I also feel almost the same. So I didn't Google the definition, but for my guess was something to do with advocate, I mean, support health, so basically supporting health of some of the people in the society. So after going through one year of Pharmacy, I think that I learnt a lot, actually, in terms of health advocacy because, as an individual myself, I am able to contribute to health advocacy with you know, as each year passed, you're closer to graduation.

You're out, you're going out to work, so if you're going out to work, that's when you can start contributing to health advocacy as an individual and maybe on the community level as well, especially when if you're working in a hospital, or maybe retail even, you still have the skills and the knowledge to help your patients.

So the question was whether I have known more about health advocacy, I would say yes, a lot especially through modules like 1150.

Interviewer:

Yeah, okay. To what factors? Let's say the factors are the curriculum, the teaching staff, CCAs, or enrichment programs. So to what factors would you attribute this change in your understanding of health advocacy to?

Student:

I mean, if I just talk about the other two. I would say I, I'm not saying that the professors and the curriculum doesn't help, on top of what [the other interviewee] said. Definitely, co-curricular activities and enrichment classes kind of help.

So CCA, they allow us to do things that we can't normally do in class or learn out of classroom. I think soft skills or like if you need to present. Okay, present is normally in class. But let's say things like if you are in the marketing society of most schools, you will know things like how to craft an actual email, how to actually reach out to sponsors to kind of contribute to your event here and there. And all these are soft, soft skills in the way. So this is just one example and also video editing and all. So definitely… oh wait, contributes to health advocacy, video-editing, no. But definitely enrichment classes, yes. So normally in pharmacy-wise, the NUS Pharmaceutical Society has been creating many different enrichment programs like Hokkien classes, Malay classes, Cantonese classes. Definitely they will attribute to health advocacy because who are the people that you face in health care in future? Definitely there are of the people who are at higher age, so they are the aging population, especially when Singapore is facing this aging population problem.

And primary care, what do we normally treat? We treat people who have chronic illnesses and these are chronic illnesses are normally found in ageing population. So definitely if we want to link them together, enrichment classes definitely will help with the communication to these ageing population. So what do they speak?

They speak dialects like Cantonese, Hokkien and also you have many different families who come. There are Malay families and they don't speak English, so that's where your Malay language skills come in and you're able to speak to them. You know it shows how proficient you are as a professional and how you can actually attribute to health advocacy, yeah, that will my answer.

Interviewer:

Yeah, okay, so from what I understand, CCAs and the enrichment programs that you've mentioned really help with your practice of health advocacy in the future or in the near future, maybe. But it doesn't really help change your understanding of health advocacy, right or like are not change but deepen your understanding of health advocacy?

Student:

I think I would say yes and no, deepen in a way it helps...

Student:

Can you repeat your question again?

Interviewer:

Yeah, okat, so from what I understand, CCAs and the enrichment programs that you've mentioned really help with your practice of health advocacy in the future or in the near future, maybe. But it doesn't really help change your understanding of health advocacy, right or like are not change but deepen your understanding of health advocacy?

Student:

I think it does, especially uh, if I may, elaborate more on why. So community service, when you interact with people from all walks of life, people that having down syndrome, especially elderly, when you go and interact with them, they tell you about your chronic illnesses here and there. So I got diabetes, etc. These are some of the things that…

If I don't step on my house and talk to these people, I wouldn't know that it's such a big thing in the society, so it kind of deepens my understanding and makes me realize more about how important health advocacy is especially for these patients. So definitely my answer will be yes.

Interviewer:

Okay yeah, the next question is, in general, what elements of teaching and learning in pharmacy curriculum like the design of the modules the projects are teaching, modes, assessment, teaching staff or learning environments or you think have an influence on the promotion of health advocacy among pharmacy undergrads, yeah?

Interviewer:

You can choose to talk about one or two or whichever you like to talk about, yeah.

Student:

So can we talk about the modules then?

Interviewer:

Said yeah, sure, like which one has an influence on the promotion of health advocacy. If none of them has any influence on the promotion of health advocacy, then you can say no and explain why.

Student:

Okay, so uh, for this question I would say I would describe two different modules that promoted health advocacy: PR1150 and GEH1049.

Student:

So these two modules have a very, very big impact on pharmacy students, and I think it was the right choice to make them compulsory modules, especially the GEH.

Especially you know, normally we can pick our GEH, but then this time round we were made to take GEH1049 and this module really showed us the figures, explain to us how important primary care is. If it were not for this module, then we wouldn't really know much about primary care.

Because it was essay question, then you have to do your research and all and you realize that as a healthcare student, you have to know the importance of it and how it's growing, how Singapore is aiming to grow this sector.

Uh, and also PR1150, a subset of date would be LPE, I think maybe [the other interviewee] wants to speak about it, but I’m not really sure. LPE is something that you get to go out there to society and interact with the patients, only one patient. Sorry, so interacting with this Indian patient and he had diabetes, he talked about how about his lifestyle changes, how he used to drink a lot, how he used to practice very bad smoking habits here and there. After he had certain conditions, he began to change his lifestyle habits and through the help of this doctor that he gave credits to, the doctor that was in charge of us.

He really was very grateful towards a doctor and how the doctor had managed to make a change in his life, and how he managed to promote health advocacy in this patient’s life. So it's something that made me realize it's not as professionals, we can actually make a difference in the lives of these individuals, and how important one impact this module has on us.

Student:

I'm so sorry if you did not 1150 lecturer.

Interviewer:

How might these elements be used to deepen the understanding of health advocacy among pharmacists?

Student:

As in improvement to the curriculum? Is it? I think they're doing a very good job already.

I would say, maybe for 1150, more Community visits. I mean, right now I believe they were yet to face the full potential of this program due to COVID. Maybe if things were in real life, maybe there will be a better learning experience for all students and especially for the fact that everything is online now, we can only contact the patient over online calls, we were able to have one group of five, one group of eight over phase two and phase three, that currently meets our learning experience by a certain amount.

So hopefully when COVID clears, we will have, especially when you don't have the personal touch like when you're together with the patient, so that makes a difference as well, so hopefully when COVID clears, things will get better. I mean, that's something that none of us can really have control of.

Interviewer:

Oh, I see. So you want it to be mentioned more specifically and a bit more explicit.

So the next question is about the elements of curriculum offered by the Department of Pharmacy. What elements of co-curriculum would you think have an influence on the promotion of health advocacy among pharmacists?

Interviewer:

Okay, so what do you think that these elements can be improved to deepen the understanding of health advocacy?

Yeah, or any co-curriculum activities that you want to see in the future?

Student:

Um, they can hold more webinars, more speakers out there to talk to us about health advocacy. I mean, these talks are very useful, normally what the pharmaceutical society created and I think a lot of our pharmacy students always go out to hear. So definitely if we have more talks, more webinars here and there, it will help to promote.

Interviewer:

I think, uh, [one interviewee] has mentioned about the integration, but I will just ask again about this. So as the new pharmacy curriculum is very much based on basic, clinical and system sciences integration, was this integration apparent to you and does it contribute to your understanding of health advocacy.

Student:

I'm a big fan of Prof. Gallagher, who is amazing person who created this curriculum, it's really very integrated.

From his curriculum, I can see a very big difference because from what I see for my senior notes, what they do is they learn any anatomy, they learn anatomy, patho anatomy, physiology, all in different modules, but in a way you don't really see the integration between them, you have to keep referring to your notes. For example, if you need a basic understanding of a certain part of the body, you need to go back to a module that maybe you took last semester or something. It's not very integrated that way because you studied last semester for as compared to what you're studying this semester.

So what we understand is that, let's say we're doing the anatomy of the eye first, then followed by that we will learn about the drugs, and then learn about how you can link a good therapy to it, how the eyes, the Physiology anatomy of the eye, then definitely you learn about the drugs that will treat the eye and then you learn different conditions as well. I mean when you link all them together, it's quite fantastic experience. And uh, how you integrate basic signs as well.

Oh okay, the foundation modules when some of us were quite lost because we are touching the very basics of the tip of the iceberg of different parts of the module. So in a way we're quite lost in there, but I think it's called foundation, so you are just supposed to know certain terms that exist here and there, so it just touches the surface.

But then the 2156 was something really very fun, something that most of us really enjoyed because we can see the integration like what I elaborated just now.

Yes, this one is basic science. Okay, I think I will stop here before I get confused about what I'm talking about. Yeah, I would say, yeah, this is what my thoughts on the integrated curriculum.

Interviewer:

And how does it contribute to your understanding of health advocacy?

Student:

If I understand what is system sciences, I think the course of a certain medication, so it's more than just the science itself, but how the health care was on a macro level.

So how it helps health advocacy. If you make the drug cheaper here in a way, then you tend to promote health because patients are able to purchase this drug able to purchase, and utilize it. So definitely it contributes to health advocacy in a way.

Student:

Okay, so for me I think I'll touch on the integration in like more of a patient care manner. So the things that we learned in the science modules we got to apply it through our patient care module, so when we actually talked to fake patient and we use the information that we learned from our other modules, so it was linked that manner and I think it was very good in my understanding of health advocacy because it not only deepens my knowledge of what we learn in the science modules because we get to actually practice it with a patient, but we also get to practice how to phrase it in a manner and actually see how we can use the knowledge to address the patients concerns and help the patient in maintaining their health.

Interviewer:

So how can the department improve on this integration?

Student:

So likewise, I would say I agree with [the other interviewee].

Uh, so since it's a new curriculum, professors are not used to the the modules being part here and there in the curriculum. So example introducing a patho teacher over to the Department of Pharmacy to teach and he doesn't … I won't blame him because it's a new curriculum, so he doesn't know why it's been taught and what is relevant in pharmacy. So... I think there are many pathology things that I would say is relevant to a medicine student, but to us, pharmacy students we never touched it before.

We never went through Physiology and all, so it's something that we are very, very unfamiliar of what it is to us.

I would say there are little holes and gaps here and there, learning what we don't know about, but then they thought that we actually know about... So I think one of the profs already addressed this point, but I just bring it up again, maybe this improvement, yeah.

Interviewer:

Okay yeah, I see.

Interviewer:

So the last two questions, what kind of modules, programs or activities related to health advocacy would you expect in your second year?

Student:

Unless I come out of the syllabus, I will say that I'm not very familiar with the term health advocacy so far in the syllabus. I always think of health advocacy as something that is more of a … I wouldn't know whether it's more for us, a very science mod.

But I think it can be linked to it, but it's like a very broad spectrum and it's not really used in our year 1 curriculum. So maybe emphasizing more on health advocacy and linking up different things to health advocacy, because I don't think we're very familiar with the term.

I think what I say is very broad, it's very general.

Interviewer:

Okay, so uh, that's about what you would expect.

I'm not sure whether it makes sense for this question, but I want to ask what kinds of the modules, programs, or activities that you would personally, like to experience. So maybe more practical activities and some case studies some scenarios for you to practice?

Student:

Yes, yes.

Student:

I will go along with that as well.

Interviewer:

Okay, sure.
