# Supplementary material for: Evaluating the internalisation of the intrinsic role of health advocacy of student pharmacists in a new integrated Bachelor of Pharmacy curriculum: a mixed-methods study
Source: BMC Med Educ. 2023 Nov 27;23:900. doi: 10.1186/s12909-023-04877-y (PMC10680209; doi:10.1186/s12909-023-04877-y)
Supplement: Supplementary file 2 — Additional file 2. [file 12909_2023_4877_MOESM2_ESM.zip › Raw Data/Post Year 1 Interview Transcripts/Post Year 1_Interviewee 5_Transcript.docx]

# Transcript of Post-Year 1 Interview with Interviewee 5

Interviewer:

Yes, so the first question is what role do you think pharmacists have to play as health advocates in Singapore society and can you give me some examples?

Student:

So what roles pharmacists play... uh, I think the first important thing for health advocacy is basically the appropriate use of medications. Teach them or educate them on how to appropriately use that medication. Like for example, you can't use medication that belongs to others, your dad or your grandfather or something. Yeah, same goals or the other way. Yeah that is one thing. Then, second thing will be the responsible use of medication in terms of antibiotics. These are two that are very related to what pharmacy does which is to advocate for the appropriate use of medication as well as other for antimicrobial stewardship. Yeah, then the third thing will be general. In general, we have to promote health in terms of promote healthy population lives, like ask them to eat healthily, exercise regularly and all.

Interviewer:

What's your definition of health advocacy?

Student:

Promote the health of the population. Health and well-being of the population, yeah.

Interviewer:

Uh, how might you see yourself as an advocate of good health in the future as a healthcare or pharmaceutical professional?

Student:

Basically I don't know whether it's a repeat of the previous question. What's the difference though?

Interviewer:

Yeah, the previous question is about pharmacists in general. The second question is about yourself.

Student:

Oh okay. I mean like so that means during practice or in general not as pharmacist, but also as like general population like how do I advise my friend and all this, is it?

Interviewer:

Uh, more like related to your job, your profession in the future.

Student:

I believe like during counseling, so this is more of like during counseling or operation in patient.

We, as much as possible, as I said in the first question, make sure that they use the drug appropriately and the drug is safe for the patient, so to reduce adverse side effects from the medication and also speedy recovery or patient. Yeah, and also like if possible for chronic diseases, of course, counsel on some non-pharmacotherapy.

Interviewer:

Okay, so that's how you see yourself in the future as a pharmacist, right? So what three characteristics best describe an effective health advocate to you?

Student:

I mean, first is knowledgeable. Second is patient. And yeah, and also understanding, yeah.

Interviewer:

May I know why you choose those three characteristics.

Student:

So first, knowledgeable, so you need to have the information about the drug and what are the non-pharmacotherapy that’s available, yeah, so knowledge is important. Then I also have mentioned patients. Yeah, so you need to have patience to explain to the patient on all these things because people may not be receptive of it, so you need to be patient and teach them step by step.

Oh understanding means, uh, sometimes you will say that you need to go exercise or you need to do certain things like you need to eat healthy diet, follow healthy play or either exercise. But a lot of times, patients may not be receptive of that because of their own lifestyles or because of their own belief, so we need to go and think from their perspectives, and after that then we can give our advice and not like: “I ask you to do this do that” but more of “Okay. I understand your needs and I try to cater my advice to your needs”.

Interviewer:

So do you feel that you have a basic understanding of what health advocacy entails? And do you feel ready to move on to the next phase?

Student:

I believe I have a basic knowledge of health advocacy.

Interviewer:

Are you ready to move on to the next phase?

Student:

What is the next phase?

Interviewer:

Depends on how you define it.

Student:

Okay, then if that’s the case, then yeah.

Interviewer:

Oh, how you define your next phase? Yeah, like I want to know in what context that you said you are ready.

Student:

So basically, next phase in terms of trying it out and practicing it.

Interviewer:

Okay.

Student:

That’s the next phase I’m thinking of.

Interviewer:

How do you see yourself in the future? Will you become a pharmacist? What would be your future job dream job?

Student:

How far in the future?

Interviewer:

Like after you graduate or maybe in 10 years.

Student:

Okay, so basically I was thinking of ... I would say I'll go to clinical practice, so clinical practice first and maybe try for four to five years. Then, I reconsider my decisions and see whether it's suitable for me or I like it or not.

If I'm still passionate, I still like it. Then of course I will stay on and pursue further studies, but if it doesn't turn out well, or I feel that I've lost motivation in that area. For me right now, the four to five years is for me to gain experience, clinical experience. In the future, I will probably use that clinical experience to basically utilize technology, so to solve problems in healthcare, yeah. So more towards like med tech and health tech, that kind of thing.

Yeah, so for me to gain the clinical context and to see what is happening, what are the problems and what are on the ground and after that develop some solutions, not necessary technological but just some solution to solve the existing problems or to improve the situation.

Interviewer:

Uh, okay, so your plan is quite clear.

Uh, so the following questions are more about the factors that drive the change in your understanding of health advocacy. Yeah, so has your understanding of health advocacy changed after your first year as a pharmacy? And if yes, to what extent has it changed?

Student:

I would say yes to some extent in terms of the drug use wise. In some lectures we did go through about antimicrobial stewardship and what role does pharmacist play in terms of antimicrobial stewardship. We work with doctors and other health care professionals to ensure the antibiotics are appropriately used, not like reduce or increase use, but appropriately used, and that's one, and secondly is about some of the lifestyle modifications that we learn in the different modules. So like for example, like acne or eye conditions, we learn about what are some of the non pharmacological management of these conditions. And learn about some of the risk factors like for example, like smoking will increase the risk of certain eye disease. And in terms of that, we know like what kind of factors we look out for in terms of when counseling the patient.

I would say that is regarding the specifics. Regarding the broad aspects, in terms of the big picture, I think I already have this idea, even before coming to uni. So uni just taught me the specifics, so I know the general concept is to do that, but I don't know how to do it. So university, maybe, yeah just it educate me on how do I do it basically. And the concept is not new to me, yeah.

Interviewer:

I see. So to what factors would you attribute this change in your understanding of health advocacy over the past year? Like considering the curriculum or the lecturers or the CCAs that you've participated in, or enrichment programs in, which one aligned with your initial understanding of health advocacy, and which one change your understanding of health advocacy? Or maybe like contributes to your understanding.

Student:

Okay contribute... I think mostly it's the curriculum wise. As I mentioned just now, it is embedded within different parts of the curriculum. I think that's good. Yeah, I believe that's only part for me.

Interviewer:

Do you participate in any relevant CCA?

Student:

No, in terms of health advocacy, no.

Interviewer:

So are they all aligned with your initial understanding of health advocacy?

Student:

Yeah I believe so, yeah.

Interviewer:

Yeah okay, so in general, what elements of teaching and learning in pharmacy curriculum, like the design of the modules or the projects or the teaching modes or assessments or lecturers or the environment that you think have an influence on the promotion of health advocacy among pharmacy undergraduates?

Student:

So what the different components ah?

Interviewer:

So which component has the like biggest effect on the promotion of health advocacy among pharmacists?

Student:

I would say is the content that is being taught, so it's the lectures.

Student:

Yeah, mostly the lectures.

Student:

Yeah, because for lectures and I mean tests, assessments, so sometimes in assessment, they ask about what some of the advice or non-pharmacological advice you can give the patients. Yeah, so I believe that's the main thing.

Interviewer:

Is the content about health advocacy in your curriculum like clear enough?

Student:

Uh, I would say it's not explicitly mentioned.

Yeah, so sometimes it may need the student to draw a bit of inference. And yeah, it's not clearly spelled out in terms of the curriculum wise.

Interviewer:

I see. How do you think these elements of teaching can be improved to deepen the understanding of health advocacy among pharmacy and undergrad?

Student:

How can be improved? So firstly, I would say is to basically explicitly state… if I remember correctly, there is only one instance whereby they mentioned about health advocacy. Yeah, only one instance and the rest of the lectures is not really explicitly mentioned.

Yeah, so maybe first they can clearly spell out what is health advocacy and the theory behind it and some of the examples because I believe everything we need to start from fundamental understanding. Yeah, so we need to make it clear in the beginning.

Interviewer:

Uh, okay.

Student:

Yeah, then that is one. Second, I mean, uh, they can have more case studies in terms of like you have real life examples on how pharmacists may be able to contribute to health advocacy. Yeah, so that would be good.

Interviewer:

So do you know about the co-curriculum in general offered by the Department of Pharmacy, like do you know about the relevant activities offered by your department?

Student:

So you mean the CCA, right?

Interviewer:

Yeah

Student:

NUSPS lor.

Interviewer:

Yes, and do you think it has an influence on the promotion of health advocacy?

Student:

So I think a lot of outreach program nowadays is being suspended or postponed or moved online, yeah everything. So I may not feel it, right now.

Interviewer:

So during this COVID period, how do you think these kinds of activities can be improved to deepen the understanding of health advocacy?

Yeah, or maybe without COVID.

Student:

Definitely with the outreach within the community, so to the community and really see what is happening on the ground. That’s one.

In terms of webinars and talks, they can also organize a few of them to specifically mention about health advocacy, so I think it's more towards public health, right?

Yeah, so I mean they can give some information about that in terms of that, yeah.

Interviewer:

Now, regarding the new pharmacy curriculum, uh, right now is very much based on basic, clinical and system sciences integration. So was the integration apparent to you and does it contribute to your understanding of health advocacy?

Student:

In terms of the integration, does the integration promote health advocacy? I would say not really. I would say what the integration does is basically make us have a better understanding of the systems itself so we can make more sense out of like for example, what we learn in like eye and skin, right?

They went through some of the basic sciences, in chemistry, in bio and yeah, then it makes us have a better understanding of what is going on in when it comes to the use of drugs for skin or eye. So that is mainly the part on it.

For the health advocacy, I would say not really because a lot of things are still very theory based, although we have some system modules in terms of the health care laws, healthcare economics, health care costs in Singapore. But this is more of a macro skills of things, which is not really related to health advocacy.

Interviewer:

How do you think the department can improve on this integration?

Student:

I don't know. I feel like this is a very broad question because which health advocacy aspect are they planning to focus on or... because you can't like you can't integrate the whole thing right?

Yeah, so that question I think I will not be able to answer for that.

Interviewer:

How can they improve on this integration to better promote health advocacy?

Student:

And not a specific aspect of health advocacy, but health advocacy in general.

Interviewer:

Yes.

Student:

Well, this one is a bit hard.

Interviewer:

Like do you expect more practical experience or like clinical experience or ...

Student:

But that is not integration, because integration means like when you learn one thing.

Uh, like we learn the foundations of thing, then you build on top of it and that is considered integration to my understanding.

Okay, so basically I was saying like integration to my knowledge is basically what you learn in one topic is related, how is it related and linked to the other topic. So that is integration.

But just now what you mentioned is in silos. So for example I want more practicals, but not that to me is not like solved integration, so I don't really know how to answer the question of integration.

How do you make like one module or like, how do you make a few modules about health advocacy linked together?

Like for integration, for basic system sciences, you learn about chemistry, bio and on top of it, you learn about the systems, so you tap on the previous knowledge to learn from chemistry and bio and you try to use that to understand the system.

But for health advocacy, I don't know how that can be done, yeah.

Interviewer:

Okay sure, no problem. So what kinds of modules or programs or activities related to health advocacy would you expect to experience in the second year?

Student:

Umm, I would say, like still basically teach us more in theory because I feel that theory is not very well-taught in year 1.

I think they should build on top of it and also at the same time, I think we will be doing more community related activities, so this is when we can apply the knowledge I've learned in health advocacy.

Interviewer:

I see. So do you expect more health advocacy-related content.

Student:

And I expect more how....

Interviewer:

Yeah, like personally, do you like it? Do you like to learn it or not?

Student:

I would say yes because it helps in terms of us as practising as a health care professional, definitely. So we can value add more in terms of our knowledge in on drugs but also in other aspects as well. So I believe that it's valuable in terms of that.
