# Supplementary material for: Evaluating the internalisation of the intrinsic role of health advocacy of student pharmacists in a new integrated Bachelor of Pharmacy curriculum: a mixed-methods study
Source: BMC Med Educ. 2023 Nov 27;23:900. doi: 10.1186/s12909-023-04877-y (PMC10680209; doi:10.1186/s12909-023-04877-y)
Supplement: Supplementary file 2 — Additional file 2. [file 12909_2023_4877_MOESM2_ESM.zip › Raw Data/Post Year 2 Interview Transcripts/Post Year 2_Interviewee 10_Transcript.docx]

# Transcript of Post-Year 2 Interview with Interviewee 10

Interviewer:

I will just see uh that the recording is done properly, that it is recording on my um computer, so just give me one minute please.

Student:

Yeap sure.

Interviewer:

Okay. Now, I am reading out the questions to the consent form, and these questions are to be read prior to the start of the interview. So number one, I acknowledge I have received a copy of the information sheet that explains the use of my interview and transcribed data in this research and I agree to participate in this research.

Student:

Yes.

Interviewer:

Okay. I understand that the audio file of my interview will be destroyed once the transcript is verified as accurate, and deletion will occur no later than two weeks from today’s date.

Student:

Yes.

Interviewer:

I understand that I can withdraw from the research prior to the completion of the interview. Once this interview is complete, there will be no way to delete the data as this interview is completely anonymous and no personal data will be recorded.

Student:

Yes.

Interviewer:

I will not have any financial benefits that result from the commercial development of this research.

Student:

Yes.

Interviewer:

I understand that the research team may use anonymized quotations from my interview in research publications and presentations.

Student:

Yes

Interviewer:

Thank you. Now, we will proceed with the interview questions.

Interviewer:

So, question one, has the Year 2 curriculum further deepened your understanding of health advocacy by pharmacists beyond the Year 1 curriculum?

Student:

I would say yes, because um… this year, we have gone through more system modules and doing that has helped us look at, um, health as a more- in a more complete manner. Looking at it beyond just the disease into the social aspects of it and uh, yes.

Interviewer:

Um, and uh, please give one thing to consider. A probe? Like, was it the curriculum, the modules, the teaching staff, co-curricular activities, enrichment programs, design of modules, project teaching modes, assessment, learning environments, like you can say any of these. So from the Year 2 curriculum, what was like, the standout element for you in health advocacy?

Student:

I would say it was the modules as well as the PECT experience that we went through.

Interviewer:

Mm hmm. Uh, can you name one in particular?

Student:

Um, okay. I’d say the modules.

Interviewer:

Okay, good thank you. So, curriculum integration. Question number two. Imagine a prospective Pharmacy student asks you to explain how the new Pharmacy program is organized. How would you explain its structure?

Student:

So, I would explain that uh, it’s built- the key differentiating factor compared to previous curriculums is that it's integrated. So, uh, our modules are grouped based on different aspects of pharmacy practice, like the content itself, which is what we learn in system modules. And then we have another module where we learnt the skills, and we can see the integration in that the skills uh, when we apply the skills that we learnt in the skills module, we use the information and content that we learnt in the systems modules. And there is an overarching module that teaches us other concepts like uh… the law regulation, ethics, and yeah, related matters.

Interviewer:

Okay, thank you. The new Pharmacy curriculum is based on the integration of basic, clinical and systems sciences. Which elements of the program best highlight the integration?

Student:

I would say the assessments because, uh for example, our skills modules where we do the…. we practice taking, you know, medication history or giving patient counselling and then the content that we learnt isn’t from that module itself. It’s from a different module so the integration is quite clear through that.

Interviewer:

Okay, um and was this integration apparent to you?

Student:

Uh, sorry?

Interviewer:

Was this integration apparent, like apparent to you?

Student:

Yes, yes it was.

Interviewer:

Yes, okay. Uh, how does the integration contribute, or not, to your understanding of health advocacy?

Student:

I think it does contribute to uh, my understanding of health advocacy because we get a more complete picture into… yeah we get a more complete picture from the beginning instead of, you know, simply focusing on certain aspects of um medicine, medicinal chemistry. Yeah, things like that.

Interviewer:

Okay. Number three. Looking ahead, what kind of modules, programs and activities related to the promotion of health advocacy would you expect to experience in your third year?

Student:

Oh dear, I think I would- I expect to do more experiential learning in the way we get to actually try out the skills and content that we learnt so we can actually apply it, instead of, you know, simply just um learning about it, yeah.

Interviewer:

So hands-on experience?

Student:

Yes, yes, hands-on experience.

Interviewer:

Okay, so we would like to accommodate that. And could you tell me from your understanding, what is health advocacy for pharmacists?

Student:

I think health advocacy is about… in a sense, promoting health to patients. So, this will take on uh, different, the different ways to go about doing it. So it could be uh, giving them advice based on their own, you know, social and other circumstances or helping them to navigate the healthcare system, making referrals if needed, uh… yeah.

Interviewer:

Correct. And how do you see health advocacy in terms of pharmacists, like what is it for pharmacists?

Student:

I think for pharmacists, a lot of it come in the counselling aspect of it when we speak to patients about their medications and we talk about uh, things like you know, non-pharmacological management and giving them advice on prevention, um you know, speaking with them, uh identifying risks and trying to tackle these health risks.

Interviewer:

Okay, thank you very much. I will now end the recording.
