# Supplementary material for: Evaluating the internalisation of the intrinsic role of health advocacy of student pharmacists in a new integrated Bachelor of Pharmacy curriculum: a mixed-methods study
Source: BMC Med Educ. 2023 Nov 27;23:900. doi: 10.1186/s12909-023-04877-y (PMC10680209; doi:10.1186/s12909-023-04877-y)
Supplement: Supplementary file 2 — Additional file 2. [file 12909_2023_4877_MOESM2_ESM.zip › Raw Data/Post Year 2 Interview Transcripts/Post Year 2_Interviewee 11_Transcript.docx]

# Transcript of Post-Year 2 Interview with Interviewee 11

Student:

Okay, okay.

Interviewer:

Okay, so I’m just going to go through the questions with you, that’s all. Um…so there's a few um questions, we’ll just go through them and um…so, the first question is, has the Year 2 curriculum further deepened your understanding of health advocacy by pharmacists beyond the Year 1 curriculum? And then if no change why? And then if yes, why? And what are the standout elements to the Year 2 curriculum?

Student:

Um wait as in… wait your question is how is the- how has the Year 2 curriculum deepened my understanding of health advocacy?

Interviewer:

Yeap, by pharmacists beyond the Year 1 curriculum.

Student:

Oh I think… I think the Year 2 curriculum is- there’s more in-depth knowledge and there’s more like integration. So like, for let’s say PR2151 and the other system modules, I think it’s great that there is integration between these modules because we get to apply our knowledge in PR2151 and try out on… uh as in we get to try like, patient communication skills together with the knowledge we learnt in the systems modules. So they’re not like, in isolation and like, separate. So in the sense like, I feel like in that way ,I can… I can help my family even like, like you know health advocacy…. like uh help them with their health and like, with all these integrated knowledge that I have learnt from… from the curriculum, yeah.

Interviewer:

Mm okay. So I guess what are like the standout elements to you about the Year 2 curriculum?

Student:

I think the… oh okay, the standout elements probably would be like… okay, the PECT in PR2150. So like, we actually get to uh, have a chance to go down for uh, to a polyclinic to be attached there for about three days. Then, we get to experience different activities that pharmacists have to carry out. And in the process, we get to learn a bit about health advocacy also, because we actually get to see the real working environment and encounter real patients, and see what their real needs are. So, I feel like PECT is very educational because when I went there, it really opened my eyes and I was like wow, like, okay so during PECT, I managed to…(inaudible) I managed to observe how a simple triage… um… eventually leads into smoking cessation so which is- which is stuff that I just learnt in PR2154 and the triage all that is- is content that is covered in PR2151. So, I feel like it's quite cool, like oh, I can see like how they are not in isolation in the- in the- in the actual work setting so I thought that was quite cool… yeah. And because- because okay in PR2151, I thought that oh okay, motivational interviewing is… by itself because it was- it was covered separately from triage. But- but like that day when I actually saw a pharmacist carrying out an actual (inaudible) like, consultation with a patient, I didn’t know that she could seamlessly transition from triage into um, smoking cessation and motivational interviewing so I thought that was quite interesting. Yeah, so I learnt that nothing is in isolation and… yeah.

Interviewer:

Mm okay. Thanks. Um, anything else to share?

Student:

Mm... I think that’s it…?

Interviewer:

Okay sure, I’ll go on to the next question then. Uh, so imagine a prospective Pharmacy student asks you to explain how the new Pharmacy program is organized. How would you explain its structure?

Student:

Okay so, how I would explain to a prospective Pharmacy student about the new Pharmacy curriculum is….so I will tell them okay, so for Year 1 Sem 1, it's mostly the basic. So they’ll teach you basic, organic- medical- medicinal chemistry and basic biology and all that. And there are some electives to take also, but that’s like, secondary to like, the learning curriculum.

So in Year 1 Sem 2, they will get like a taste of the first systems module, which is PR2156, which is the eye and skin. Uh yeah, so then that is where the professors will start to link the content taught in the systems modules to the clinical skills modules which is PR1151 and… and some of the things… and yeah, basically, we get to tie our content to patient communication. And then the patient communication module there is… we get to learn compounding and patient communication skills as well as drug information. So, drug information is like we… we have to search for information quickly to answer the drug-related enquiries of, let’s say, doctors or patients et cetera, yeah.

Then, in Year 2, it will mostly be the systems modules. So, it will be the remaining systems, the CVS, GI, um, renal and hepatic and respiratory, and as well, there will be a continuation of the- of the clinical- of the patient skills module so that will be more in-depth. Oh, and then there’s another module which covers um, what is expected of a pharmacist and pharmacist values all that, so that will be in both Year 1 and Year 2, they will be year-long modules. The Year 2 one will be more in-depth knowledge like oh ethics, et cetera, et cetera. So by the time Year 2 Sem 2 is over, all the systems modules will be over, uh wait… they will be mostly over.

Then in Year 3, there will be the final year… sort of final year project because the professors moved it to Year 3. Then there will be a module on infection which is the antibiotics all that, and the…and there will be some…I think some brain… like yeah brain medicine all that and yeah so the- so the final year project will be year-long. Then there will be some, if I’m not wrong, there’s some patient skills also. So basically yeah, it’s just increasing complexity when- as we go up and it’s like, kind of like step by step, like increasing complexity.

Yeah, so by the time Year 4... Year 4 Sem 1 the entire Sem 1 will be, will be compulsory attachment. So I think half of it is industry and half of it is in the community pharmacy. Then Year 4 Sem 2 we will come back to do some other modules before we graduate. Then, after we graduate, if you wish to get your license, you have to undergo an additional 6 months of pre-reg at the end at uh, at an approved establishment by SPC and do pre-reg there for 6 months before you can get your license. Uh yeah, and there are some tests also something like that? That’s how I would explain the curriculum.

Interviewer:

Okay, thanks. Uh okay, so next question is, the new Pharmacy curriculum is based on the integration of basic, clinical and system sciences. Which elements of the program best highlight the integration, and was this integration apparent to you? Just let me know if you need me to repeat it.

Student:

Oh okay. Uh so your question is, which part of the curriculum highlights uh… integration between the systems modules and the clinical aspect the most?

Interviewer:

Uh, which elements of the program best highlight the integration. So, because the pharmacy curriculum is based on the integration of basic, clinical and system sciences, so which elements of the program best highlight the integration.

Student:

Oh okay… I think the- the aspect of like the program, the portion that best highlights the integration would be- probably be in PR2151. Because yeah, as I mentioned just now um the professors will teach clinical skills like oh history taking, uh triage and uh that kind of like motivational interviewing and um… um yeah like pharmaceutical care plan all that so… then that is where they will- they will tie in to the- to the systems modules.

So let's say we just finished learning about the gastrointestinal medicine… like… last semester so in PR2151 they will… the- the questions that they set, be it for test or- or like for class, it will be related to the gastrointestinal tract and the medicines so we get a- we have a chance to like consolidate our information and apply it to the patients skills. Yeah, so we can apply it to like, the triage like what are the red flags to look out for in the gastrointestinal system, and like for the medicine, oh what kind of medicine is suitable for this kind of condition, and then we try to communicate it to the (inaudible) patient by applying the skill learnt in PR2151. So I think that’s where the integration is most apparent.

And probably in PR2150, it will be PECT I guess… yeah because we get to go out and observe new pharmacists at work. And yeah, we will need to apply our systems knowledge as well yeah, so we won’t go there and like waste time, you know. Because like, we kind of know what’s going on before we go there, yeah. If not we will just be observing the patient and be like, wait, what’s going on? Yeah, so I guess that’s also where the integration is, is um apparent yeah, so we- we go to the site with- well-equipped with knowledge of patient communication and systems uh systems modules knowledge so… so when we go there, we get to learn like… oh this is how the pharmacists um communicate certain- certain um information to patients then we get to learn along the way so- so our PECT experience will be more fruitful. Yeah, so I guess those are the two elements.

Interviewer:

Hmm okay. So was this integration apparent to you?

Student:

I think the integration was quite apparent, because especially in PR2151, they like to integrate the knowledge we have learnt, be it PR1151, like those two modules, they like to integrate both- uh they like to integrate the systems modules knowledge inside, so it’s quite apparent. Then in PECT, yeah because I went to the site and I tried to apply what I had learnt, so I felt like I had, I benefitted from the experience. Yeah, so this integration is quite apparent.

Interviewer:

Mm. Alright thanks. So the next question is how does the integration contribute or not, uh, to your understanding of health advocacy?

Student:

Mm… I think the integration has- has probably contributed to my knowledge of health advocacy because- because we get to- so first we learn the systems knowledge, so the pharmacotherapy, the symptoms- signs and symptoms, red flags within the systems modules.

Then we go to the- to the skills- to the patients communication and skills module where we… where this knowledge is integrated into communicating such information to patients or trying to get some information from patients to help us come up with uh, what probably the patient is experiencing so we can suggest the appropriate pharmacological management, non-pharmacological management and monitoring and follow-up, et cetera, to the patient so in the pharmaceutical care plan. Yeah so I feel like this has probably contributed to health advocacy because I believe health advocacy is a full picture so this patient… (inaudible). We want to make their life better, so like it’s an interplay of many factors, so of course we need to have very strong systems… systems modules knowledge.

And we also need to have- we need to have good patient communication skills so that we get the information across to patients in a way such that they understand and they’re more likely to adhere to their medicine, so it's very important in health advocacy, yeah, as it can to maintain- it can help to probably improve their symptoms and probably improve their quality of life yeah depending on the patient… mm.

Interviewer:

Okay, thank you. Um, so there's two more questions. The next one says, looking ahead, what kind of modules, programs and activities related to health advocacy would you expect to experience in your third year?

S: Mm… what kind of modules do I expect… mm… I think, wait, hang on, I need to look at the Year 3 modules. I think, yeah let me Google.

Interviewer:

Oh wait, I guess it’s more… what would you expect to experience?

Student:

Okay, Year 3… there will be the… okay I guess I can talk about the FYP, like the discovery project… so it’s a year-long project. I think probably can experience health advocacy inside, because need to come up with a presentation on a…on a certain topic that we choose that is related to health and we need to tell our idea to the professors, like something like that…like elevator pitch so I guess that process we will get to learn more about health advocacy? Like how our project can improve certain things? Something like that? Yeah, and I guess like the infection control modules… yeah all that.

Interviewer:

Okay, thanks. Um, alright so the last question says, what kinds of modules, programs and activities related to the promotion of health advocacy would you personally like to see or experience? And then give some examples.

Student:

Oh, as in, as in it might not exist now, right? Like, it's just what I hope to see?

Interviewer:

Mm. What would you personally like to see.

Student:

Um, or like it can be modules or like just short programs?

Interviewer:

Um, so the question just says modules, programs and activities and to give some examples so… yeah.

Student:

Mm… I think… I think probably… mm… maybe some of the activities I would like to see would probably be like… mm… okay, so like the academic- wait, no no, not the academic committee, one of the sub committees in NUSPS they hold like those befriending sessions with the elderly or (inaudible). I think probably there can be more such activities which Pharmacy students can join because, because okay I personally joined one of them- one such activity before so it was befriending with the elderly. Uh, yeah at some centre, then I realized that actually they, they need like how to say like, accompaniment? Like someone to talk to them… yeah, because the patient we talked to, he expressed that he felt very happy to be able to like, talk to young people like us, yeah, and he felt very happy to meet us. So we like, we would ask him about his health, and then uh yeah we’d do some activities with him. So, then on the whole, he felt happier, so I think probably we can have more activities like that, especially the number of elderly with like, diseases, are rising and they probably need some like, emotional support? Like, because some of them, they might not have children, or like the children are too busy to like, take care of them. So I feel like, in a sense like, health advocacy because, these elderly they need people to ask them like, oh how are you today? Are you taking your medicine properly? And et cetera, et cetera. So yeah, it's part of health advocacy because we get to accompany them and like make them feel happier… Yeah so they, so it's kind of like mental-health related? So it's also like- and also because we get to remind them if they forget to take a dose or something, and then probably can like give them some advice on how to like manage their condition, so overall, I think can have more of such activities in general, yeah, so like more people can take part… mm …

Interviewer:

Mm. Okay, thank you so much. Uh, okay I think that’s the end of it, yeah that’s the end of the questions, so let me just stop the recording.
