# Supplementary material for: Evaluating the internalisation of the intrinsic role of health advocacy of student pharmacists in a new integrated Bachelor of Pharmacy curriculum: a mixed-methods study
Source: BMC Med Educ. 2023 Nov 27;23:900. doi: 10.1186/s12909-023-04877-y (PMC10680209; doi:10.1186/s12909-023-04877-y)
Supplement: Supplementary file 2 — Additional file 2. [file 12909_2023_4877_MOESM2_ESM.zip › Raw Data/Post Year 2 Interview Transcripts/Post Year 2_Interviewee 6_Transcript.docx]

# Transcript of Post-Year 2 Interview with Interviewee 6

Student:

Hello, Dr Emina.

Interviewer:

Can you hear me?

Student:

Now I can hear you.

Interviewer:

Okay, how are you doing today? Um, can I ask you to unmute yourself? Now, I cannot hear you again.

Student:

Oh, sorry. Uh… ya. I’m good, thank you.

Interviewer:

Yes, happy to have you here for this interview, and that you responded, this is really nice.

Student:

I’m happy to be here.

Interviewer:

Yes, um… when you’re ready, I will just ask you to uh… to review the informed consent. And I will read it out to you, and you just say yes if you are okay with this.

Student:

Yes, sure.

Interviewer:

Uh, as you know this interview is being recorded, so um… I will just read out the questions to you and feel free to answer whatever you think, um yes, any answer is appreciated.

So, um, please, just to acknowledge, I acknowledge I have received a copy of the information sheet that explains the use of my interview and transcribed data in the research, and I agree to participate in the research.

Student:

Yeap, I acknowledge, and I agree.

Interviewer:

I understand that the audio file of my interview will be destroyed once the transcript is verified as accurate, and deletion will occur no later than two weeks from today’s date.

Student:

Yeap, I understand, and I agree.

Interviewer:

I understand that I can withdraw from the research prior to the completion of the interview. Once the interview has been completed, there will be no way to delete the data as the interview is completely anonymous and no personal data will be recorded.

Student:

Yeap, I understand, and I agree.

Interviewer:

Okay. I will not have any financial benefits that result from the commercial development from this research.

Student:

I understand, and I agree.

Interviewer:

And I understand the research may use anonymized quotations from my interview in research publications and presentations.

Student:

Yes, I understand, and I agree.

Interviewer:

So, okay. So, we can proceed now with the interview?

Student:

Okay.

Interviewer:

Okay, just give me a moment.

Interviewer:

So, I would like to ask you a few questions regarding your health advocacy. And, um… yes, you have watched the videos in your class?

Student:

Yeah.

Interviewer:

Before, yeah.

Student:

Oh, the module.

Interviewer:

Okay. So, this is um, so to say, these are questions for the interview like post-scenario. And my first question to you will be, has the Year 2 curriculum further deepened your understanding of health advocacy by pharmacists beyond the Year 1 curriculum?

Student:

Um, personally in my opinion is it?

Interviewer:

Mm hmm.

Student:

I would, I would say so. I think it has taught me, especially like PR2150 and PR2151, uh it teach me how to deal with a greater profile of patients compared to last year.

Interviewer:

Okay. And uh… Why and what are the elements to the Year 2 curriculum? So, things to consider like curriculum modules, teaching staff, co-curricular activities, enrichment programs, design of modules, project teaching modes, assessment, learning environments, physical, virtual etc. So, what are the standout elements to the Year 2 curriculum in your opinion?

Student:

Um, I feel that the face-to-face graded assignments in 2151 are probably the most useful ones, because I think there is a difference between online classes and face-to-face. So, um, especially when we are face-to-face, then we are able to… to… when we have the role-playing and then the profs act as the patients right? And then we are able to generally observe how they interact with the patients in the scenarios.

And I feel that another element would be how they introduced to us, section by section, of interacting with patients. So in Year 1 Semester 1, we had only history taking. And then, um, in the first semester of this year, we had… like they added on diagnosing, minor ailments as well. And then, this year, we… this semester, we had suggesting treatment for minor ailments. So that step-by-step gradual exposure, I would say, has also helped me to… to be able to learn at a better rate.

Interviewer:

Right, thank you. So, we move on to question two, which is curriculum integration.

Student:

Yeap.

Interviewer:

Please imagine a prospective Pharmacy student who asked you to explain how the new Pharmacy programme is organized. How would you explain its structure in your words?

Student:

Uh… I would say… so, we have um… a bunch of Pharmacy modules that we have to take. And then, one… every semester, we have about one or two that are focused on systems, and then another one that is focused on patient interactions, and then another one which is focused on the laws, principles, and I would say, ethics of um… Pharmacy.

And, I think at any point in time in each semester, all of the… all of the separated modules are sort of collaborated in a sense, where for instance, in this semester, we are currently learning about the respiratory system. So, in the module about patient interaction, most of the interactions will also be about the respiratory system. So, we are sort of revising for one module at the same time as we are learning the other module, or as we are practicing the other module. Which, I would say, has been beneficial for me lah. Because it feels like I am studying for one big subject as opposed to studying for three separate things at once.

Interviewer:

Yeah, very good. Yeah, thank you.

Uh, the new Pharmacy curriculum is based on the integration of basic, clinical, and system sciences. Which element of the programme best highlights the integration? And was this integration apparent to you?

Student:

I… I think definitely the integration is most apparent in um… the PR2151. Because, when we… in the systems module, we just learn how to, for example, administer certain drugs and stuff. And then, when we go on to the patient interaction module, then we actually get to instruct the patients how to do that. And then, the module coordinators for that particular module also use the notes from the systems module, so that is when the integration is very apparent, I think, to everybody, because the notes are taken directly from the other module.

Interviewer:

Mm hmm.

Student:

Yeah, I think so. That is when the integration is clearest to all of us.

Interviewer:

Okay. And how does the integration contribute to your understanding of health advocacy?

Student:

Mm hmm… I would say, we don’t view it as a separate… separate part of being a pharmacist. For instance, if it was like a separate module with no integration, then you’d see the systems as one part of being a pharmacist, and then patient interaction as another part, which we may not be able to link up the two. But with the integration of, and the application of the systems modules into the patient interaction modules right, we are able to see certain things in the systems module that we can use in our health… in advocating for good health among our patients. So, I would say that is something that has helped my health advocacy.

Interviewer:

Okay. And um… Third question. Looking ahead, what kind of modules, programmes, and activities related to the promotion of health advocacy, would you expect to experience in your third year?

Student:

Mm hmm, I think for the third year, we are also working on our FYP. So, I’m not sure… I’m not sure if the experience will be quite the same as this year, or the integration would be… say, two modules combined into one, that kind of thing.

Interviewer:

Okay.

Student:

Um… But I would expect more of the same thing, more of honing our skills with patient interaction, and then probably we will have more complex patient profiles integrating all the different systems that we have learnt in the past, and we will continue to learn in the future, I would say.

Interviewer:

Okay. And um… What would you like, personally, to see? Give some examples, can you give me some examples on what programmes and activities related to promotion of health advocacy would you like to see in your third year?

Student:

Um… I don’t know if it is possible because of Covid. But it would be nice to be able to uh, I don’t know… uh… walk around the neighbourhood or pharmacies or clinics, and then be able to talk to patients or people in general about their health. Um… and maybe in relation to the module that we are learning, so maybe about the respiratory system, then we could be going to some smoking cessation clinics, and then maybe watching how pharmacists deal with these patients, or talking to… to real-life patients, instead of just the profs acting as patients.

Interviewer:

Yeah, like you know, real-life experience. I agree. I think it would be possible… yeah soon.

Student:

Hopefully.

Interviewer:

Yeah, hopefully soon. I agree. Exactly.

Um… And may I just ask you one question that I am personally interested in? So, what do you think… are pharmacists… um… what’s their role in the health advocacy process? Where could you… do they have an active role as a profession? And how can they influence the society or promote better structures, um… healthcare structures, and help the healthcare?

Student:

Um, before I joined Pharmacy, I didn’t really know what the role of a pharmacist was in like, a Singaporean society, or like a community. So I think, probably, in the future, in order for pharmacists to be able to play a bigger role, the society has to know that pharmacists do exist and that we are capable of giving um… information that is credible to them. Yeap.

I feel that pharmacists can… do play a huge role in health advocacy actually. Because when patients go to the pharmacy and stuff, they may not necessarily meet the doctors all the time right?

Interviewer:

Yes, correct.

Student:

Um, they may have to wait for the appointments, and sometimes, even when they pick up medications, they don’t see the doctor either. They just go straight to the pharmacy.

Interviewer:

Correct.

Student:

So, um… I think these are important junctures to which they need reminders to take care of their health, or certain conditions, or keep an eye out for some alarm symptoms. And that pharmacists are the only ones able to give them, because they are the only ones who are interacting with these patients at this point in time.

Interviewer:

Yeah. And it’s not just the individual patients, but also many patients… to reach out to many patients, to the society, to even go to the higher levels. You know, also to influence the politics, and to advocate for better healthcare. Yeah, very important role, I agree with you… I agree.

Interviewer:

So, um… thank you very much. I will just finish recording now.
