# Supplementary material for: Evaluating the internalisation of the intrinsic role of health advocacy of student pharmacists in a new integrated Bachelor of Pharmacy curriculum: a mixed-methods study
Source: BMC Med Educ. 2023 Nov 27;23:900. doi: 10.1186/s12909-023-04877-y (PMC10680209; doi:10.1186/s12909-023-04877-y)
Supplement: Supplementary file 2 — Additional file 2. [file 12909_2023_4877_MOESM2_ESM.zip › Raw Data/Post Year 2 Interview Transcripts/Post Year 2_Interviewee 7_Transcript.docx]

# Transcript of Post-Year 2 Interview with Interviewee 7

Interviewer:

Okay, so um… today, we are having this interview. And I would like to ask you these following questions prior to the start of this interview.

So, I acknowledge I have received a copy of the information sheet that explains the use of my interview and transcribed data in this research, and I agree to participate in this research.

Student:

Yes.

Interviewer:

Okay. I understand that the audio file of the interview will be destroyed once the transcript is verified as accurate, and deletion will occur no later than two weeks from today’s date.

Student:

Yes.

Interviewer:

I understand that I can withdraw from the research prior to the completion of the interview. Once the interview is complete, there will be no way to delete the data as the interview is completely anonymous and no personal data will be recorded.

Student:

Yes.

Interviewer:

I will not have any financial benefits that result from the commercial development of this research.

Student:

Yes.

Interviewer:

I understand that the research team may use anonymized quotations from my interview in research publication and presentations.

Student:

Yes.

Interviewer:

Okay, so you agreed to these terms before?

Student:

Yes.

Interviewer:

Thank you, and now we can proceed with the interview. So, just give me a second. And um… I will read out the first question to you.

So, the first question would be, has the Year 2 curriculum further deepened your understanding of health advocacy by pharmacists beyond the Year 1 curriculum?

Student:

I would say yes, because we had the opportunity to go to the health site, which is polyclinics. So we got to experience what pharmacists actually do, and… as well as the other healthcare professionals.

Interviewer:

Okay. And could you please quote the standout elements to the Year 2 curriculum which helped you? Like things to consider would be curriculum modules, teaching staff, co-curricular activities, enrichment programmes, design of modules, and so on. What was the standout element in the… in the curriculum, Year 2?

Student:

Yes. I think there are two things. One of them is the… the site visits I mentioned just now.

Interviewer:

Mm hmm, okay.

Student:

And the other one is the module, which is the cardiovascular module, as it was…

Interviewer:

Yeah. What was specific about the cardiovascular module?

Student:

I thought that it was really well-planned. So it follows a certain theme, which starts from the anatomy and physiology, which followed to the hypertension, to the heart failure, as well as arrhythmia.

Interviewer:

So it has… it has good structure for you. So you could…

Student:

Yeap. So it was easy to follow as well.

Interviewer:

Easy to follow. Okay thank you, that is very valuable.

So, um… about the curriculum integration, please think. Imagine a prospective Pharmacy student asks you how to explain the new pharmacy programme, how is it organized? How would you explain its structure?

Student:

I would say that now, we focus more on the clinicals, because it is what our healthcare structure is meant to be in the future. But other than that, we also focus on the clinical sciences, and we also like… the pharmaceutical science and pharmaceutical technology as well. So we are pretty versatile in that sense, rather than just focusing on the clinicals itself.

Interviewer:

Okay. The new pharmacy curriculum is based on the integration of basic, clinical and system sciences. Which elements of the programme best highlights the integration? Was this integration apparent to you?

Student:

The systems sciences part was quite… was the main thing. Because we follow… every half the semester, we will follow the different body systems. So like last semester, we did gastrointestinal system and the cardiovascular system. And now we are doing the... the respiratory system as well as the hepatic and renal system. So, there is this structure that is in this curriculum.

Interviewer:

Okay. How does the integration contribute, or not, to your understanding of health advocacy?

Student:

There are certain elements like, which I won’t say is there in every module, but let’s say for this respiratory system, there is this smoking cessation lecture that teaches us what smoking does to the body and the harmful effects. Then, we can further understand what we can do as healthcare professionals to try to... to get smokers to try to stop smoking because it is better for them to stop, due to many financial costs, even health costs as well. So it is more like a preventive medicine in a sense.

Interviewer:

Okay. And looking ahead, what kinds of modules, programmes, and activities related to the promotion of health advocacy would you expect to experience in your Year 3? In your third year, what kinds of modules, programmes, and activities would you personally like to see or experience? Give some examples if you can, for Year 3.

Student:

For me, personally, I would like to have more on-site visits. Because I am more of a hands-on learner, so it is better for me to see in person, as well as to learn. And other than that, maybe more about preventive medicine. Because this is where I feel that healthcare is more important, because that is before the start of… before the start of a disease, which can be prevented.

Interviewer:

Mm hmm.

Student:

Yeah.

Interviewer:

Yeah, very good. And then I would just like to ask you one additional question regarding the advocacy... health advocacy as a pharmacist. So, how do you see this for the profession of Pharmacy, once you become a pharmacist. What can you do, how do you see the health advocacy as a role of a pharmacist?

Student:

For me, it is mainly uh… for me, it is mainly health literacy, as well as preventive medicine. Because, like health literacy can help to improve knowledge about the drug and the side effects as well. And because we are pharmacists, we should have the most knowledge about the drugs. So, as such, because some drugs have very serious side effects, and that should be made known to the patients as well, so that they do not abuse the medicines.

Interviewer:

Uh, so do you see it more on a personal level? Like on an individual level?

Student:

It can be based on both. Like, for example, during dispensing, that is a personal level. And maybe, using the public sites, maybe like the government websites, that could help to expand to the public as well, so we can reach out to more people, and maybe can tell them what does this medicine do, and what are the possible side effects and what they should look out for.

Interviewer:

Yeah, very good. Yeah, that would be all. Thank you so much. I will stop recording now.

Student:

Yeah, no problem.
