# Supplementary material for: Evaluating the internalisation of the intrinsic role of health advocacy of student pharmacists in a new integrated Bachelor of Pharmacy curriculum: a mixed-methods study
Source: BMC Med Educ. 2023 Nov 27;23:900. doi: 10.1186/s12909-023-04877-y (PMC10680209; doi:10.1186/s12909-023-04877-y)
Supplement: Supplementary file 2 — Additional file 2. [file 12909_2023_4877_MOESM2_ESM.zip › Raw Data/Post Year 2 Interview Transcripts/Post Year 2_Interviewee 8_Transcript.docx]

# Transcript of Post-Year 2 Interview with Interviewee 8

Interviewer:

Okay. Hello. Welcome to this interview. Um… I will read out the consent statements prior to the start of this interview.

So, I acknowledge and I have received a copy of the information sheet that explains the use of my interview and transcribed data in this research, and I agree to participate in this research.

Student:

Yes.

Interviewer:

I understand that the audio file of my interview will be destroyed once my transcript is verified as accurate, and deletion will occur no later than two weeks from today’s date.

Student:

Yes.

Interviewer:

I understand that I can withdraw from the research prior to the completion of the interview. Once the interview is complete, there will be no way to delete the data as the interview is completely anonymous, and no personal data will be recorded.

Student:

Yes.

Interviewer:

I will not have any financial benefits that result from the commercial development of this research.

Student:

Yes.

Interviewer:

I understand the research team may use anonymized quotations from my interview in research publications and presentations.

Student:

Yes.

Interviewer:

Okay, thank you very much. And now, we will proceed to the interview questions. Just give me a second, I will open the interview questions.

So, my question number one. Has the Year 2 curriculum further deepened your understanding of health advocacy by pharmacists beyond the Year 1 curriculum? If no, why? And if yes, why, and what are the standout elements to the Year 2 curriculum?

Student:

Uh… can I just confirm that health advocacy is basically something related to how people have access to health services?

Interviewer:

Yes. Did you have health advocacy in Year 1?

Student:

Uh… I’m not too sure about that. But I, uh… I believe I did.

Interviewer:

Okay. And uh… the Year 2 curriculum, you had cases. I guess you did it with Dr Leroy?

Student:

Uh, yes.

Interviewer:

So he presented some cases from the newspaper, and some videos, I guess. So you did discuss something like this, if I recall correctly.

Student:

Yes, there were certain case studies on how accessible healthcare was for Asian populations and et cetera.

Interviewer:

Exactly, yes.

So, did you think you have deepened your understanding of health advocacy by these studies, case studies?

Student:

Uh, yes, I certainly did, yeah.

Interviewer:

OK. And, do you recall are there any standout elements to the Year 2 curriculum? Like, things to consider are curriculum modules, teaching staff that stood out, co-curricular activities, enrichment programmes, design of modules, projects, teaching modes, assessments, learning environments. Anything physical and virtual that you can think of.

Student:

Uh… unfortunately, there is nothing standout that comes to mind.

Interviewer:

Mm hmm.

Student:

Yeah. But, I did learn… um, it is not really a one big chunk, but rather, it is a lot of small information from different modules that come together and actually tell me more about how health advocacy is like in Singapore.

Interviewer:

Okay, thank you.

So, my second question would be curriculum integration. Imagine a prospective pharmacy student. The student asks you to explain how the new pharmacy programme is organized. How would you explain its structure?

Student:

Uh… I would say that it is organized like a ladder. Yeah. So it’s like uh… the foundation is built in Year 1 during like uh… 1152 and 1153. Then uh… there are then different systems modules coming in. And even on these system modules, there are more system modules that are built on it, yeah.

Interviewer:

Okay. Question B. The new pharmacy curriculum is based on the integration of basic, clinical and system sciences. Which element of the programme best highlights the integration? Was this integration apparent to you?

Student:

Uh… I would say the integration would… most… I feel like uh, the integration is only appearing most recently, yeah. So uh, because you know recently the respiratory module? Yeah, so the respiratory module had certain elements of CVS stuff in it. Yeah, so that’s where I feel where all the integration comes in. I feel that in Year 1 and Year 2 Sem 1, the integration isn’t that obvious. So I think the full integration will only be apparent maybe in Year 3 and Year 4 as well.

Interviewer:

Okay, when you… as you progress.

Student:

Yeah, yeah.

Interviewer:

Okay. How does the integration contribute, or not, to your understanding of health advocacy?

Student:

Uh, sorry?

Interviewer:

How does this integration, integrated curriculum, contribute to your understanding of health advocacy? Or, how does it not contribute?

Student:

Uh, I think in addition to the system modules, there are other modules such as the 1150, 2150, then uh, 1151 and 2151, that actually talk about all these other side issues, other than the system sciences. So like the more, uh… social issues, the more like uh, issues regarding like inequality, pharmacoeconomics, pharm law, all these. Yeah, I think all these actually uh, develops us holistically as pharmacists, other than just the system sciences like how we treat patients, choice of therapy, et cetera, et cetera.

Interviewer:

Yes, very good. And question number three. Looking ahead, what kinds of modules, programmes and activities related to the promotion of health advocacy would you expect to experience in your third year?

Student:

Uh, in third year, maybe uh… since Year 2 has covered aging population, perhaps we will see more of at risk populations? Maybe those patients with like AIDS, or like uh, disabled people, or like uh, troubled youth, those kind. Yeah, maybe that will be an interesting element to look at as well regarding health advocacy.

Interviewer:

Okay. And what kinds of modules, programmes and activities related to the promotion of health advocacy would you personally like to see or experience?

Student:

Uh, I think definitely uh, for health advocacy, I think uh… the best way of learning is really hands-on. So like you know, visiting these people, you know actively reaching out to them, perhaps having like a uh, having like a… assignment. You know like how in 1150, there is this six hours of CIP contribution?

Interviewer:

Yeah.

Student:

Yeah. So maybe like, something like that in Year 3. But something that is more regulated and more strict. Because I feel that the six hours was very easy to circumvent. So maybe a structural collaboration between like the department and certain beneficiaries would really help to put things regarding health advocacy into perspective as well.

Interviewer:

Okay, great. And one last question of mine. How do you see health advocacy in the pharmacist profession? Do you have any thoughts about it?

Student:

Oh, like from the perspective of a pharmacist? Or…?

Interviewer:

Yes. Once you are a pharmacist, what would health advocacy be to you?

Student:

Uh, I mean… I think it depends on what kind of pharmacist I will be, I guess. Maybe as like a retail pharmacist, it will be slightly different from how I view it as a hospital pharmacist?

Interviewer:

Mm hmm.

Student:

Because maybe like, hospital pharmacists, there are a structural… there are already structures in place on how to help all these underprivileged, how access of care should be granted. You know, because each hospital has their own system, and there are national programmes such as MediSave, MediCare, et cetera, et cetera, that actually regulate access to care.

But maybe for retail pharmacist, it is a bit harder, and maybe it will go a bit more personal. Because as retail pharmacists, you know, patients come to us with their problems, and they tell us like what kinds of problems they are facing. And we can’t really interfere since we are not like directly, directly giving care to them. Yeah, so I think it will really depend on what kind of pharmacist you are.

Interviewer:

Yeah, what you do, yeah. Do you see health advocacy as also some kind of promotion for pharmacists?

Student:

Oh, so like, oh... oh... oh... oh, like pharmacists will use this to further the profession, or?

Interviewer:

Yes. Could you imagine that this is also health advocacy?

Student:

Uh, yeah, I think definitely. Since uh, I mean uh, maybe uh, the physicians themselves might be a bit busy taking care of the root issue. And pharmacists can play a part. But I guess other healthcare workers, they also tackle that part, right? Like social workers, et cetera, et cetera.

Interviewer:

Yeah.

Student:

So, uh, yeah. But I do think there’s a part to be played by pharmacists, I guess?

Interviewer:

Mm hmm.

Student:

Yeah.

Interviewer:

Okay, good. Thank you very much. So I will stop recording now.
