# Supplementary material for: Evaluating the internalisation of the intrinsic role of health advocacy of student pharmacists in a new integrated Bachelor of Pharmacy curriculum: a mixed-methods study
Source: BMC Med Educ. 2023 Nov 27;23:900. doi: 10.1186/s12909-023-04877-y (PMC10680209; doi:10.1186/s12909-023-04877-y)
Supplement: Supplementary file 2 — Additional file 2. [file 12909_2023_4877_MOESM2_ESM.zip › Raw Data/Post Year 2 Interview Transcripts/Post Year 2_Interviewee 9_Transcript.docx]

# Transcript of Post-Year 2 Interview with Interviewee 9

Interviewer:

Okay. So I’m just going to run through some questions with you, okay?

Student:

Mm hmm.

Interviewer:

Okay. So has the Year 2 curriculum further deepened your understanding of health advocacy by Pharmacists beyond the Year 1 curriculum? If no change, why? And then if yes, why and what are the stand-out elements to the Year 2 curriculum?

Student:

Erm… can I ask if this is with respect to um… 2150 or as a… like um, all the modules?

Interviewer:

Mm… I’m- I’m not exactly sure I’m guessing the whole curriculum though.

Student:

I see… okay hmm. Um, I guess I feel like um yes, definitely because um… maybe I’ll just go through the different modules and what my takeaways in terms of um, health advocacy?

Interviewer:

Okay!

Student:

Um, I think that um, specifically I think 2153 the… our cardiovascular module um helped to… because a lot of the informa-, like um, what um… the profs actually went through was a lot of clinical reasoning, um not sure how that um… exactly links to health advocacy but I feel like because of being able to learn that skill um, I am better able to um… provide um… more um… better able to make judgements in the future as a future healthcare provider and so, getting that insight from the whole module, I think was um… quite impactful and something that I have gained um in the aspect of being a health advocate in the future and as of now as well.

Yeah, and then um… for 2150 I feel that a lot of the content we covered this year was about the um… healthcare system in Singapore so… I guess we have learnt how um… because they can be quite complicated with the whole networking and all? So um, the fact that we are being educated on that and how the systems are in place and what is the rationale behind these systems, I think um… puts a lot of what we see in practice to perspective. Yeah and um, it also helps me to be aware of the current healthcare system and as well to educate my friends and family who might not be aware of this knowledge, so I think in those two ways specifically that erm…my- the role of the healthcare- health advocate has been uh, impressed upon us even more so than in Year 1.

Interviewer:

Okay, thanks. Um okay, so the next question is imagine a prospective Pharmacy student asks you to explain how the new Pharmacy program is organized. How would you explain its structure?

Student:

Um so I would say that it is a combination of three essential bodies right, so the first part is the knowledge part which is um… which is given to us through content through our content modules. Like, um the different system modules like 2154 and 2155 right, those are the content.

But it doesn’t just stop there right, um it actually integrates with the skills as well, which is the second main component and that is why we have the modules such as um… 2150, I mean sorry, 2151 and 1151 where we hone the skills of um being a future pharmacist? Right, so it actually ties in a lot with the knowledge that we have learnt so we have to tap on the content we learnt from content modules and then apply it into skills right that’s where we practice.

And then lastly, it’s not just about knowledge and skills, it's also about the (inaudible) that we do and the kind of professionalism that we carry as future professionals. And um, so there’s modules such as 2150 and 1150 to hone those and to um, help us shape our attitudes and um build professionalism among students as well and with the different programs such as PECT now brought earlier into our curriculum, we’re able to get a more tandem(???) experience and practical view of what we will be doing in the future. Yeap, I would say it's a very practical and um holistic experience… yeap the new change.

Interviewer:

Mm okay thank you. Okay, so the next question is the new Pharmacy curriculum is based on an integration of basic, clinical and system sciences. Which element of the program best highlights the integration and was this integration apparent to you?

Student:

Okay, um I’m so sorry, I only just remember three parts.

Interviewer:

Oh yeah yeah, no problem.

Student:

Okay, maybe um… can I clarify the difference between the basic and the system sciences again?

Interviewer:

Um… I think these yeah… I’m not exactly sure but I think the basic maybe… is referring to um… the less like system-based sciences, so maybe more of the…

Student:

Foundation?

Interviewer:

Yes… foundational… I think so.

Student:

Okay (inaudible). The profs got introduce before but…

Interviewer:

I’m guessing that is what it is referring to.

Student:

I guess… um… which ones stood out to me the most?

Interviewer:

Which elements of the program best highlights the integration.

Student:

I would say, firstly the clinical as well as the systems, I guess. Um so maybe, I’ll share more about the systems first so… I guess because now the modules are constructed in a way that um, it’s everything about the system that is being taught in one module rather than having it split up into topics for example pharmacochemistry as one module, I mean um medicinal chemistry one module, pharmacokinetics another module, you know, um it allows us to… having this um sort of module system allows us to be able to… like I said, have a holistic view and able to integrate the information that we learnt very well. So, not only do we learn about the anatomy of the uh… our bodies of that particular system, we learn about the diseases we can easily make the connections as well as the therapeutics that are involved…help us integrate our knowledge better and because of that I think it is very… um…. I’d say for me it is easier to comprehend but I can’t speak for everybody yeah.

So that’s what I really appreciate about the new curriculum and um as well as the clinical side because um I guess the curriculum has now been shifted to really promote this agenda of um, you know, getting a degree in pharmacy sort of…they’re sort of promoting this um career in the healthcare rather than in all the other sectors per se. So yeah, they’re really training your clinical skills through the introduction of things, elements such as the (???) exam? And um, all these skills lab sessions? Which may not have been um so… emphasized in the previous curriculums. Yeap, so I think those two elements stick out the most to me and I appreciate the department for um actually rolling it out in such uh effective methods.

Interviewer:

Okay, thanks. Um so I guess the second half of that question was just uh, was this integration apparent to you?

Student:

Mm yes, I would say yes even though I’m not too sure what the differences between basic and system sciences are right now. But um, yes I can see that there is a very big integration that is seen throughout the module… throughout the academic journey.

Interviewer:

Okay, sounds good. Um, how does the integration contribute to your understanding of health advocacy?

Student:

Um…

Interviewer:

Or not. It says how does the integration contribute or not to your understanding of health advocacy.

Student:

Alright, um definitely my answer is yes. It helps, um I guess, um because, um it really as I said, it integrates the context. So if I take an example the um… let’s say the respiratory mod, um we’re able to see from… the uh so uh maybe a subset of a role as a health advocate is also to promote health right? So um… in the sense that when we learn about smoking cessation and um how to conduct the services through our content module 2154, as well as our skills module 2151, we’re able to see the connection by the skills and also to see how this can actually um impact other- you know our patients or just anyone who is um, who uh, wants to um you know, quit smoking and we’re able to use this knowledge to help them on this healthcare journey with them…

I think um… the curriculum, the integrated manner helps us to see this connection very clearly, which I think if it wasn’t integrated in this way, we wouldn’t be able to see such a close relation and the outcome is that we appreciate how important these services are and we are more empowered to continue being health advocated even before we are officially pharmacists and registered to practice, but we also take note of that right now.

Interviewer:

Okay thanks. Alright so the last question says… looking at what kind of modules, programs and activities related to health advocacy would you expect to experience in your third year?

Student:

Mm.. the truth is that I’m not too sure what they have planned out for us as well. So um, yeah the thing about being the new batch is that um, in the new curriculum, is that we are the first to try everything. It's a lot of trial and error but I’m glad that we are able to provide our feedback and the department is able to offer their um, what they can based on our feedback.

I guess one aspect is that uh, I think they’re really… probably more PECT rotations and basically more time out of the school context and really just appreciating what pharmacists do… yeah… I think that to me plays the biggest role in helping to shape what I view about pharmacists and how um pharmacists can actually um really make a huge impact in this healthcare system that we’re in and continue to educate the general public.

I think it would be great if we could have more opportunities to do more outreach programs to the general public… you know um, just like I think in the past there were like medical reconciliation events that you could volunteer at. Yeah, I mean there still are now, but because of the Covid situation… that would be good for our exposure and to learn more skills, even more than what um the curriculum is teaching right now to becoming an even better health advocate than what we’re learning now.

Interviewer:

Okay, thanks. I know um you mentioned one thing that you thought you would like to see, um that you personally would like to see. Is there anything else in terms of programs, modules or activities in relation to the promotion of health advocacy that you would like to see or experience?

Student:

Um… I guess um…. not that I can think of in my head, yeah. But um maybe I’ll just mention that um there is a lot of um… there’s a lot of emphasis on… in Singapore and worldwide as well, on mental health, so I think that is something we can continue to explore because I don’t think we have really touched that yet. I’m not sure exactly how pharmacists will contribute in that area but I think it’s something in the coming year that we could continue to look out for. And also, it’s part of this whole concept of health advocacy which is often overlooked, because they usually look at the chronic diseases, acute diseases and not really so much mental health so… something to think about, yeah. That’s all.

Interviewer:

Mm okay cool, thank you. Um alright, thank you for your responses. That's all the questions we have so that’s it.

Student:

Oh, okay.

Interviewer:

Thank you so much for your time.

Student:

Thank you, bye bye.

Interviewer:

Have a nice afternoon. Thank you, bye bye.

Student:

You too, bye bye.
